# Short and dysfunctional telomeres protect from allergen‐induced airway inflammation

**DOI:** 10.1111/acel.13352

**Published:** 2021-05-04

**Authors:** Sergio Piñeiro‐Hermida, Paula Martínez, Maria A. Blasco

**Affiliations:** ^1^ Telomeres and Telomerase Group Molecular Oncology Program Spanish National Cancer Centre (CNIO) Madrid Spain

**Keywords:** 6‐thio‐dG, allergy, house dust mite (HDM), telomerase, telomeres

## Abstract

Asthma is a chronic inflammatory disease affecting 300 million people worldwide. As telomere shortening is a well‐established hallmark of aging and that asthma incidence decreases with age, here we aimed to study the role of short telomeres in asthma pathobiology. To this end, wild‐type and telomerase‐deficient mice with short telomeres (third‐generation (G3 *Tert* ^−/−^ mice)) were challenged with intranasal house dust mite (HDM) extract. We also challenged with HDM wild‐type mice in which we induced a telomere dysfunction by the administration of 6‐thio‐2´‐deoxyguanosine (6‐thio‐dG). Following HDM exposure, G3 *Tert* ^−/−^ and 6‐thio‐dG treated mice exhibited attenuated eosinophil counts and presence of hematopoietic stem cells in the bone marrow, as well as lower levels of IgE and circulating eosinophils. Accordingly, both G3 *Tert* ^−/−^ and 6‐thio‐dG treated wild‐type mice displayed reduced airway hyperresponsiveness (AHR), as indicated by decreased airway remodeling and allergic airway inflammation markers in the lung. Furthermore, G3 *Tert* ^−/−^ and 6‐thio‐dG treated mice showed lower differentiation of Club cells, attenuating goblet cell hyperplasia. Club cells of G3 *Tert* ^−/−^ and 6‐thio‐dG treated mice displayed increased DNA damage and senescence and reduced proliferation. Thus, short/dysfunctional telomeres play a protective role in murine asthma by impeding both AHR and mucus secretion after HDM exposure. Therefore, our findings imply that telomeres play a relevant role in allergen‐induced airway inflammation.

## Graphical abstract

Diminished expression of SOX2 in airway epithelial Club cells of mice with short and dysfunctional telomeres prevents differentiation of Club cells to goblet cells, reducing mucus secretion and eosinophil recruitment to the lung upon house dust mite (HDM)‐induced allergy. Club cells of mice with short/dysfunctional telomeres also displayed increased DNA damage (γ‐H2AX) and senescence (p21), as well as decreased proliferation (Ki67). Moreover, decreased bone marrow myelopoiesis (CD34) in these mouse models also contributes to reduced presence of eosinophils in the lung.

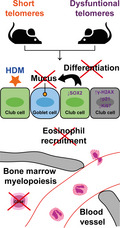

## 1 INTRODUCTION

Approximately 300 million people worldwide suffer from asthma with at least 250000 deaths attributed to the disease each year, which results in substantial morbidity and annual healthcare expenditure (Dharmage et al., 2019; Fahy, 2015). Asthma is a chronic inflammatory disease of conducting airways characterized by airway hyperresponsiveness (AHR) and airflow obstruction. The pathological changes that occur in the airway epithelium and submucosa known as “airway remodeling,” include subepithelial fibrosis, smooth muscle hypertrophy, and goblet cell hyperplasia. Inhaled β2‐adrenergic and leukotriene receptor agonists, glucocorticoids, and IgE‐directed therapies continue to be the main treatment for individuals with asthma (Fahy, 2015; Lambrecht & Hammad, 2015).

House dust mite (HDM) allergens are the most important source of mite‐related allergens. HDM exhibits a complex mixture of molecules and activators of the innate immune system serving as adjuvants to disrupt intercellular tight junctions leading to cytokine, chemokine, and growth factor production and cellular influx, as well as airway remodeling and mucus hypersecretion (Buday & Plevkova, 2014; Calderón et al., 2015; Gregory & Lloyd, 2011). The airway epithelium is an essential controller of inflammatory, immune, and regenerative responses in asthma. In response to HDM allergens, the airway epithelium secretes fluids, antimicrobial proteins, and mucins which together with Club cells represent a major part of the immunomodulatory barrier of the airway epithelium (Lambrecht & Hammad, 2012; Whitsett & Alenghat, 2015).

Telomeres are protective structures localized at the ends of eukaryotic chromosomes, which are essential for chromosome stability (Blackburn, 2001). In mammals, telomeric DNA consists of TTAGGG tandem repeats bound by a 6‐protein complex known as shelterin (De Lange, 2005; Liu et al., 2004). With each cell division, telomeres shorten due to the incomplete replication of chromosome ends, a phenomenon known as the ‘‘end‐replication’’ problem (Olovnikov, 1973; Watson, 1972). Telomere shortening can be compensated through the *de novo* addition of telomeric repeats onto chromosome ends by telomerase, a reverse transcriptase composed of a catalytic subunit (TERT) and an RNA component (Terc), used as a template for telomere elongation (Greider & Blackburn, 1985).

Telomerase (TERT) mutations result in extremely short telomeres and have been associated with the clinical manifestations of several respiratory pathologies including idiopathic pulmonary fibrosis and chronic obstructive pulmonary disease (Alder et al., 2008; Stanley et al., 2015). Although the implication of short telomeres in asthma is unknown, a shorter peripheral leukocyte telomere length was reported in asthmatic patients (Belsky et al., 2014; Kyoh et al., 2013), but additional mechanistic and association studies are needed to confirm this hypothesis. On the other hand, telomerase‐deficient mice were reported to display attenuated anaphylactic responses indicating a possible role of short telomeres in protection of allergic reactions (Ujike‐Asai et al., 2007).

Although at the moment different therapeutic approaches for telomerase‐based treatment of cancer have been investigated without success, telomeres remain as potential therapeutic targets in oncology (Bejarano et al., 2017; García‐Beccaria et al., 2015; Martínez & Blasco, 2017). Despite asthma incidence decreases progressively with age (Dharmage et al., 2019; Pakkasela et al., 2020), a role for telomeres in asthma has not been yet defined.

Here, we aim to investigate the implication of short and dysfunctional telomeres in asthma pathobiology. To address this, *Tert* ^+/+^ and G3 *Tert* ^−/−^ mice were challenged with HDM extract to induce allergic airway inflammation (Piñeiro‐Hermida, Alfaro‐Arnedo, et al., 2017; Piñeiro‐Hermida, Gregory, et al., 2017). In addition, telomere dysfunction was also induced in wild‐type mice by using the nucleoside analog 6‐thio‐2´‐deoxyguanosine (6‐thio‐dG), which is incorporated into telomeric DNA by telomerase. The telomere sequence TTAGGG is then modified at guanine bases leading to telomere dysfunction, genomic instability, and cell death (Mender et al., 2015). We show that short/dysfunctional telomeres play a protective role against allergen‐induced airway inflammation.

## 2 RESULTS

### 2.1 Mice with short telomeres show an attenuated allergy in response to HDM challenge

**FIGURE 1:**
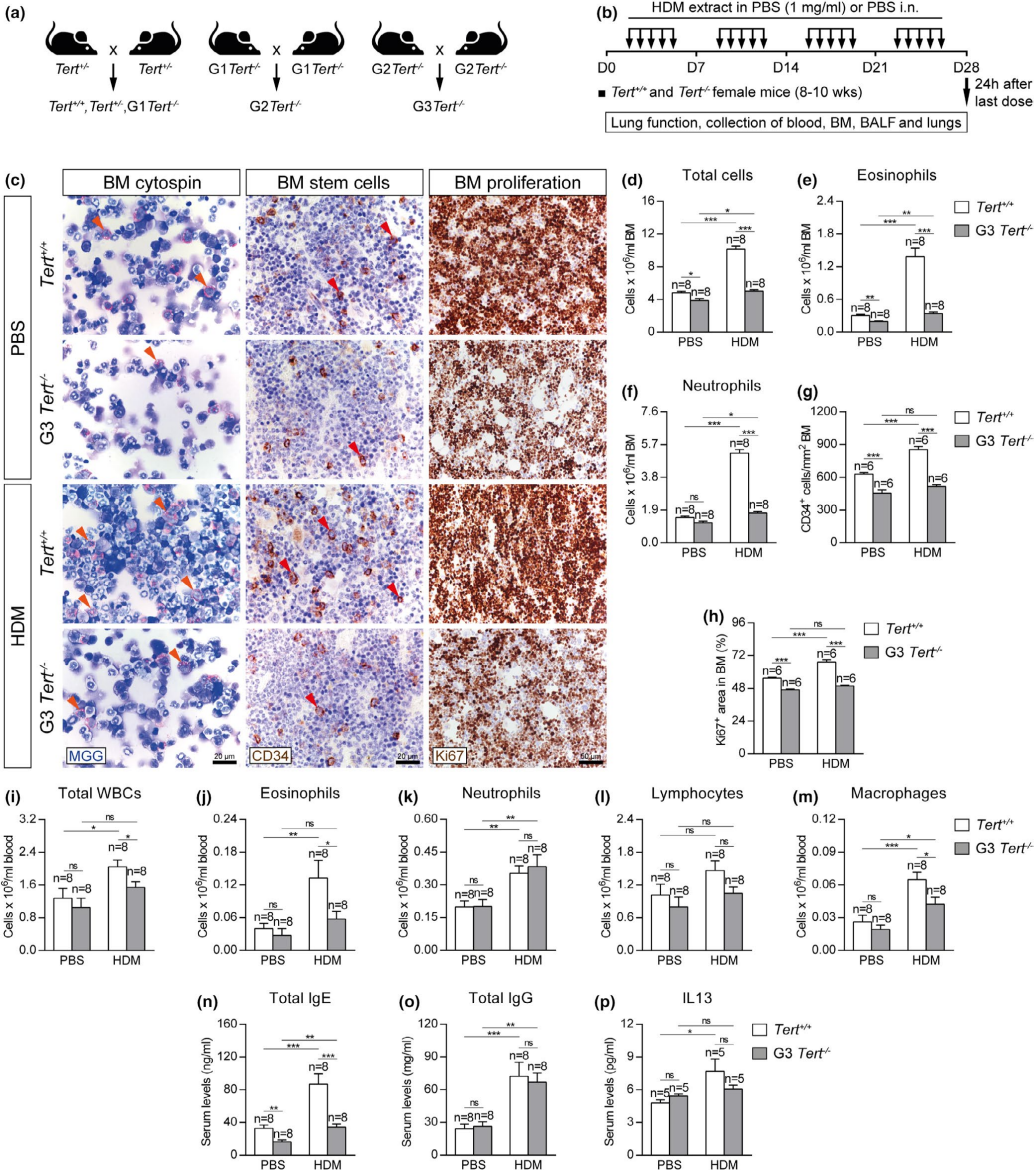
Telomerase deficiency results in decreased eosinophils, hematopoietic stem, and proliferating cells in the bone marrow and diminished serum IgE and IL13 levels and circulating eosinophils after HDM‐induced allergy. a‐b, Generation of *Tert* ^+/+^ and G3 *Tert* ^−/−^ mice and protocol for chronic exposure to house dust mite (HDM). a, Heterozygous *Tert* ^+/−^ mice were crossed to obtain *Tert* ^+/+^ and G1 *Tert* ^−/−^ mice, and successive crosses between G1 *Tert* ^−/−^ and then G2 *Tert* ^−/−^ were set to generate G3 *Tert* ^−/−^ mice. b, Eight‐ to 10‐week‐old female *Tert* ^+/+^ and G3 *Tert* ^−/−^ mice were challenged by intranasal administration of 20 μg of house dust mite (HDM) extract in 20 μl of phosphate‐buffered saline (PBS) (1 mg/ml) or equal volume of PBS under inhaled isoflurane anesthesia, five days a week during four weeks. Lung function assessment and blood, bone marrow (BM), BALF, and lungs were collected 24 h after the last exposure to HDM on day (D) 28. c, Representative images showing cytospin preparations (May‐Grünwald Giemsa (MGG) (orange arrowheads indicate eosinophils)) (left) and immunostainings for CD34^+^ hematopoietic stem cells (brown) (red arrowheads) (center) and Ki67^+^ proliferating cells (brown) (right) in bone marrow sections from *Tert* ^+/+^ and G3 *Tert* ^−/−^ mice. Quantification of total cells (d), eosinophis (e), neutrophils (f), CD34^+^ hematopoietic stem cells (g) and Ki67^+^ proliferating cells (h) in bone marrow sections from *Tert* ^+/+^ and G3 *Tert* ^−/−^ mice. Quantification of total white blood cells (i), eosinophils (j), neutrophils (k), lymphocytes (l), and macrophages (m) in peripheral blood, and Total IgE (n), Total IgG (o) and IL13 (p) levels in serum from *Tert* ^+/+^ and G3 *Tert* ^−/−^ mice. Quantifications in bone marrow cytospins and sections were evaluated in 4 different fields in a random way. Data are expressed as mean ±SEM. *p < 0.05; **p < 0.01; ***p < 0.001 (Dunn–Sidak multiple comparison test). The number of mice is indicated in each case

Since the role of telomere length in asthma is unknown, we aimed to investigate its implication in asthma pathobiology. For that purpose, wild‐type mice (*Tert* ^+/+^), as well as third generation (G3) telomerase‐deficient mice with short telomeres (G3 *Tert* ^−/−^) (Blasco et al., 1997) were intranasally challenged with house dust mite (HDM), extract to induce allergic airway inflammation (Piñeiro‐Hermida, Alfaro‐Arnedo, et al., 2017; Piñeiro‐Hermida, Gregory, et al., 2017) (Figure 1a,b). First, bone marrow cellularity as determined by May‐Grünwald Giemsa staining, number of hematopoietic stem cells as determined by CD34‐positive staining, and proliferation as determined by Ki67 staining, were evaluated in the bone marrow of *Tert* ^+/+^ and G3 *Tert* ^−/−^ mice (Figure 1c‐h). G3 *Tert* ^−/−^ mice showed less total, eosinophil, and neutrophil counts compared to wild‐type mice in the control PBS‐treated mice (Figure 1c‐f). Interestingly, upon HDM exposure, *Tert* ^+/+^ mice showed a further increase in total, neutrophil and eosinophil counts, while G3 *Tert* ^−/−^ mice did not show such an increase (Figure 1c‐f).

We also observed a decrease in the number of CD34^+^ hematopoietic stem cells (CD34^+^ cells/mm^2^) in G3 *Tert* ^−/−^ mice compared to wild‐type mice in control PBS‐treated conditions (Figure 1c,g). Again, we observed that CD34^+^ hematopoietic stem cells increased in wild‐type mice after exposure to HDM, while this increase was not seen in G3 *Tert* ^−/−^ mice (Figure 1c,g). A similar scenario was seen in the number of Ki67^+^ proliferating cells (percentage of Ki67^+^ area) in the bone marrow that increased in the wild‐type mice upon HDM exposure but not in the G3 *Tert* ^−/−^ mice (Figure 1c, h).

Next, we determined blood cell counts in the different mouse cohorts. We found that HDM‐treated G3 *Tert* ^−/−^ mice showed significantly lower numbers of total white blood cells, as well as of eosinophils and macrophages compared to similarly treated wild‐type mice (Figure 1i‐m).

Next, we assessed the levels of IgE and IL13, commonly used as clinical diagnostic biomarkers of allergy, in serum from the different mouse cohorts (Agache et al., 2016; Coverstone et al., 2020). In control conditions, G3 *Tert* ^−/−^ mice showed lower serum IgE levels than wild‐type mice (Figure 1n). Upon HDM exposure, IgE levels increased to higher levels in wild‐type mice compared to G3 *Tert* ^−/−^ mice (Figure 1n). In addition, serum IgG and IL13 levels were similarly increased after HDM exposure in wild‐type and G3 *Tert* ^−/−^ mice (Figure 1o‐p).

### 2.2 Mice with short telomeres show an attenuated pulmonary pathology upon HDM exposure

**FIGURE 2:**
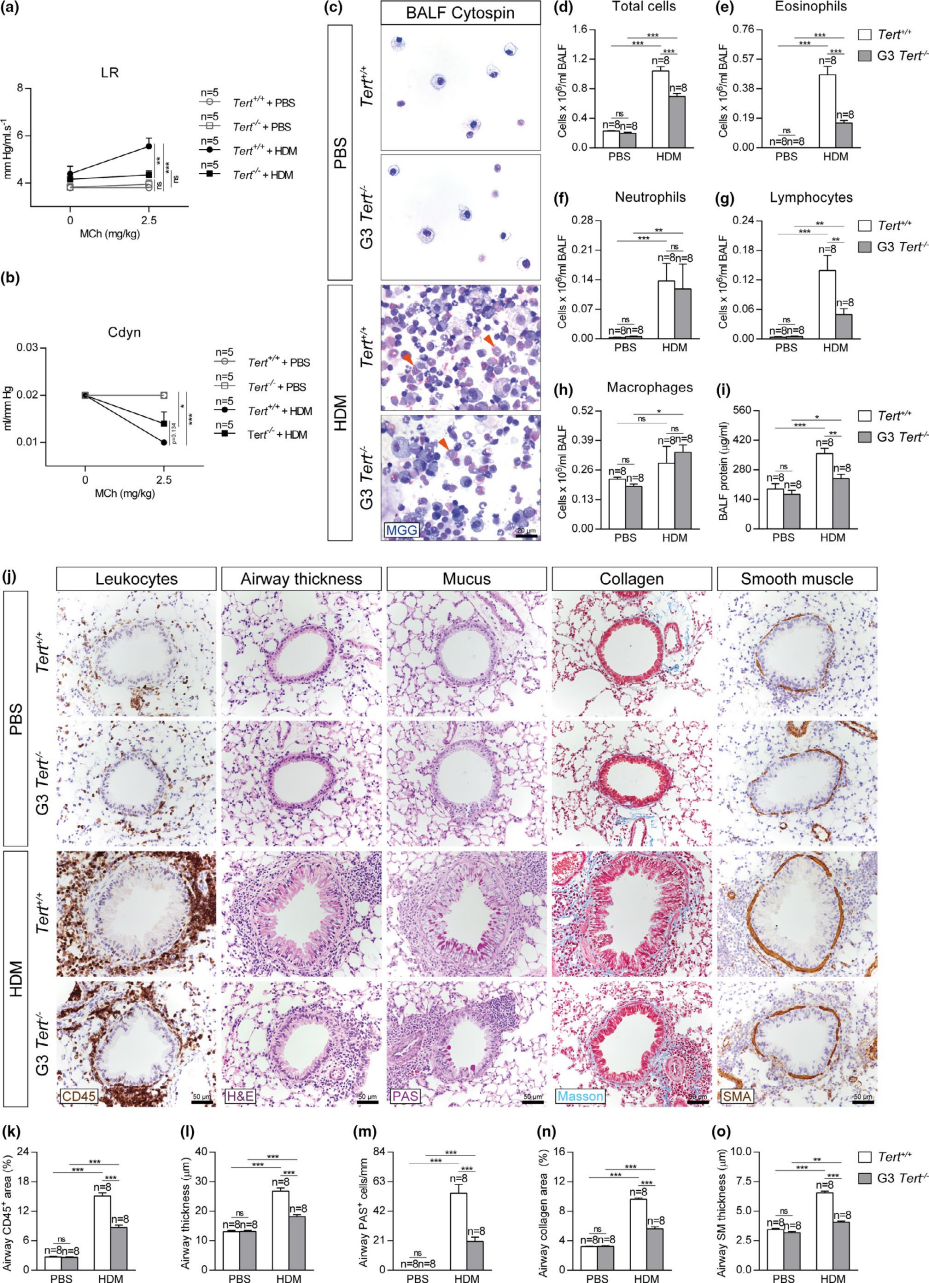
Telomerase deficiency attenuates AHR, eosinophil, and lymphocyte counts in BALF and reduces leukocyte presence and airway remodeling after HDM exposure. Quantification of lung resistance (LR) (a) and dynamic compliance (Cdyn) (b) to methacholine (MCh) evaluated by plethysmography in *Tert* ^+/+^ and G3 *Tert* ^−/−^ mice. Representative BALF cytospin preparations (May‐Grünwald Giemsa (MGG) (orange arrowheads indicate eosinophils)) (c) and quantification of total (d) and differential BALF cell counts for eosinophils (e), neutrophils (f), lymphocytes (g), and macrophages (h), and total protein concentration in BALF (i) of *Tert* ^+/+^ and G3 *Tert* ^−/−^ mice. Representative images of proximal airways showing CD45 (brown) and H&E (left), PAS (pink) (center) and Masson (blue), and SMA (brown) (right) stainings and immunostainings (j) and quantification of airway CD45^+^ area (leukocytes) (k), airway thickness (l), airway PAS^+^ cells (m), airway collagen area (n) and airway smooth muscle (SM) thickness (o) in lung sections from *Tert* ^+/+^ and G3 *Tert* ^−/−^ mice. Quantifications in lung sections were performed in 4 different bronchi in a random way. Data are expressed as mean ±SEM. *p < 0.05; **p < 0.01; ***p < 0.001 (Dunn‐Sidak multiple comparison test). The number of mice is indicated in each case

In order to address the effect of short telomeres on lung pathology following HDM‐induced allergy, we evaluated airway hyperresponsiveness (AHR) to methacholine by plethysmography, a well‐established technique to measure lung function in mouse models of allergic airway inflammation (Verheijden et al., 2014). HDM‐challenged *Tert* ^+/+^ mice displayed AHR as indicated by increased lung resistance (LR) and decreased dynamic compliance (Cdyn) (Figure 2a,b). In contrast, lung functional responses in HDM‐treated G3 *Tert* ^−/−^ mice remained unaffected and similar to PBS‐treated controls (Figure 2a,b). Thus, lung resistance (LR) was significantly decreased in G3 *Tert* ^−/−^ mice compared to wild‐type mice following HDM exposure. Also, G3 *Tert* ^−/−^ mice showed increased dynamic compliance (Cdyn) compared to wild‐type mice after HDM exposure, although the difference did not reach statistical significance (Figure 2a,b).

As G3 *Tert* ^−/−^ mice showed a better lung function following HDM‐induced allergy compared to wild‐type mice, we next assessed lung pathology by determining lung cellularity and total protein levels in bronchoalveolar lavage fluid (BALF) (Figure 2c,i). We found that HDM‐challenged *Tert* ^+/+^ mice showed significantly higher cell counts for eosinophils and lymphocytes compared to similarly treated G3 *Tert* ^−/−^ mice in BALF (Figure 2c‐h). Furthermore, *Tert* ^+/+^ mice showed significantly higher protein levels in BALF compared to similarly treated G3 *Tert* ^−/−^ mice (Figure 2i).

Next, we addressed whether the presence of short telomeres protected against HDM‐mediated allergic airway inflammation by determining airway thickness (µm), presence of airway leucocytes (airway CD45^+^ area, (%)), presence of mucus‐producing cells (PAS^+^ cells/mm), airway collagen deposition (airway collagen area (%)), and airway smooth muscle (SM) thickness (µm) (Figure 2j‐o). Assessment of these airway remodeling indicators revealed increased values in HDM‐challenged lungs of *Tert* ^+/+^ and G3 *Tert* ^−/−^ mice compared to their PBS controls and although airway remodeling was significantly higher in *Tert* ^+/+^ mice compared to G3 *Tert* ^−/−^ mice, this increment was milder in *Tert* ^−/−^ mice following HDM exposure (Figure 2j‐o).

**FIGURE 3:**
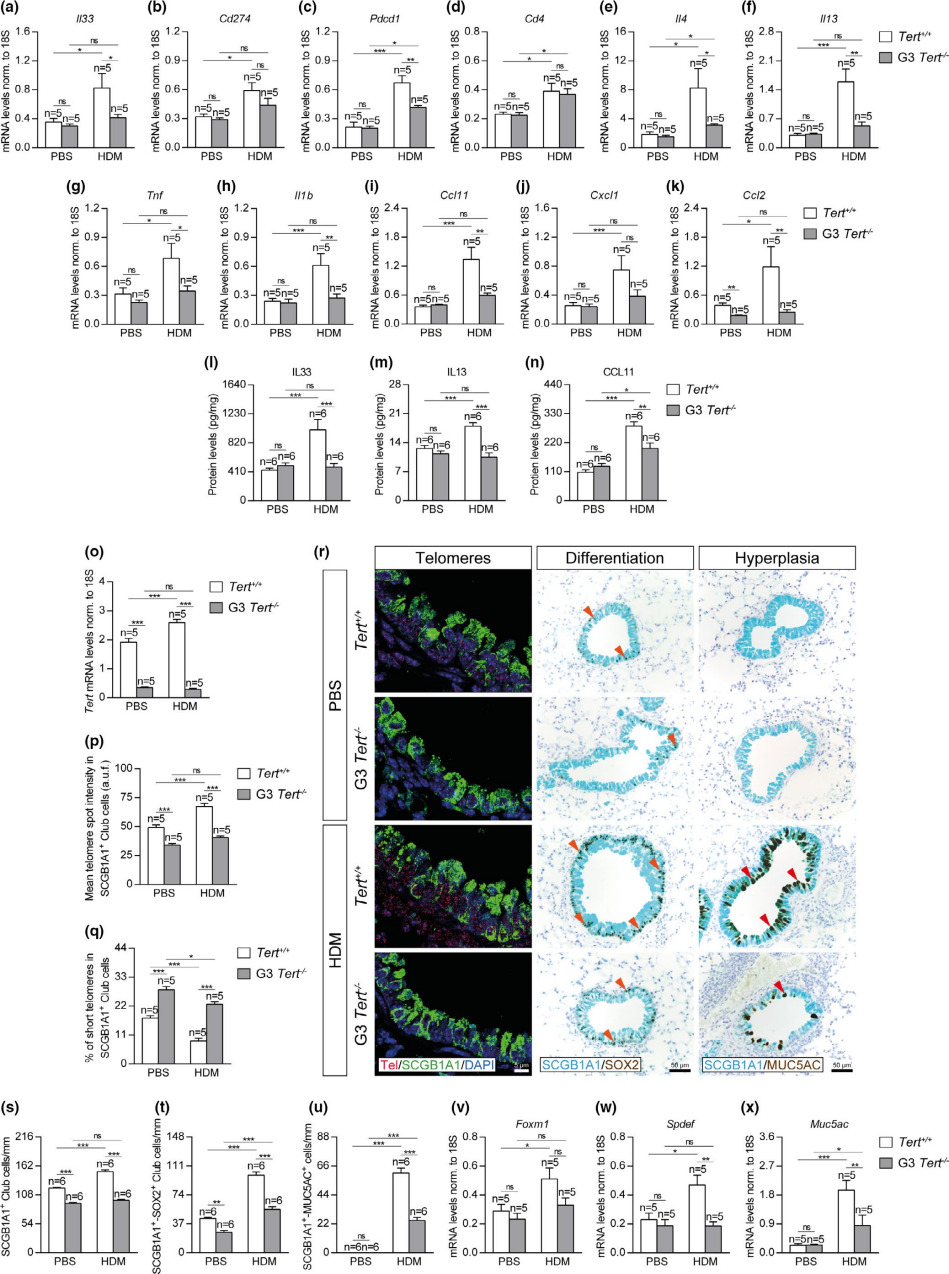
Telomerase deficiency reduces the expression of allergic markers, prevents telomere elongation and differentiation in Club cells, and attenuates goblet cell hyperplasia after HDM exposure. (a‐k) Lung tissue mRNA expression levels of *Il33* (dendritic cell activation) (a), *Cd274* (PD‐L1) (b) and *Pdcd1* (PD‐1) (c) (Immune checkpoint, T‐cell activation), *Cd4* (T‐cell marker) (d), *Il4* (e) and *Il13* (f) (Th2 cytokines), *Tnf* (g) and *Il1b* (h) (Th1 cytokines), *Ccl11* (eosinophil chemotaxis) (i), *Cxcl1* (neutrophil chemotaxis) (j) and *Ccl2* (macrophage chemotaxis) (k) in *Tert* ^+/+^ and G3 *Tert* ^−/−^ mice. Total lung mRNA levels were normalized to 18S expression. l‐n, IL33, IL13, and CCL11 protein levels in lung homogenates from *Tert* ^+/+^ and G3 *Tert* ^−/−^ mice. (o‐q) Lung tissue mRNA expression levels of *Tert* normalized to 18S expression (o), quantification of mean telomere fluorescence (mean telomere spot intensity) (p), and percentage of short telomeres (q) in Club cells corresponding to the 20^th^ percentile of the fluorescence intensity values of controls (PBS‐challenged *Tert* ^+/+^ mice) in *Tert* ^+/+^ and G3 *Tert* ^−/−^ mice. r, Representative images of proximal airways showing a Q‐FISH for telomere spot fluorescence in Club cells (Cy3Tel probe (red), SCGB1A1^+^ cells (green), and nuclei stained with DAPI (blue)) (left) and representative immunostainings for SCGB1A1 (blue) and SOX2 (brown; orange arrowheads indicate double SCGB1A1^+^‐SOX2^+^ Club cells) (center) and SCGB1A1 (blue), and MUC5AC (brown; red arrowheads indicate double SCGB1A1^+^‐MUC5AC^+^ cells) (right) in lung sections from *Tert* ^+/+^ and G3 *Tert* ^−/−^ mice. Quantification of SCGB1A1^+^ (s) or double SCGB1A1^+^‐SOX2^+^ Club cells (t) and SCGB1A1^+^‐MUC5AC^+^ cells (u) per epithelium length, and total lung mRNA expression levels of the goblet cell hyperplasia markers *Foxm1* (v), *Spdef* (w) and *Muc5ac* (x) normalized to 18S expression in *Tert* ^+/+^ and G3 *Tert* ^−/−^ mice. Quantifications in lung sections were performed in 4 different bronchi in a random way. Data are expressed as mean ±SEM. *p < 0.05; **p < 0.01; ***p < 0.001 (Dunn–Sidak multiple comparison test). The number of mice is indicated in each case

In order to further study how short telomeres protect from HDM‐induced allergy, we studied the expression of several allergic airway inflammation markers such as *Il33* (dendritic cell activation), *Cd274* (PD‐L1), and *Pdcd1* (PD‐1) (Immune checkpoint, T‐cell activation), *Cd4* (T‐cell marker), *Il4* and *Il13* (Th2 cytokines), *Tnf* and *Il1b* (Th1 cytokines), *Ccl11* (eosinophil chemotaxis), *Cxcl1* (neutrophil chemotaxis), and *Ccl2* (macrophage chemotaxis) (Figure 3a‐k). In all cases, except for *Cd274*, *Cd4*, and *Cxcl1*, the mRNA expression levels of allergic airway inflammation markers induced by HDM were significantly higher in *Tert* ^+/+^ mice than in G3 *Tert* ^−/−^ mice (Figure 3a‐k). Indeed, in marked contrast to wild‐type mice which showed an increased expression of all inflammation markers upon HDM exposure, G3 *Tert* ^−/−^ mice exposed to HDM did not show increased expression of *Il33*, *Cd274*, *Il13*, *Tnf*, *Il1b*, *Ccl11*, *Cxcl1*, and *Ccl2* markers (Figure 3a‐k). These findings were also supported by analysis of IL33, Il13, and CCL11 protein levels in lung homogenates. In particular, IL33, Il13, and CCL11 protein levels were significantly higher in *Tert* ^+/+^ mice following HDM‐induced allergy, whereas G3 *Tert* ^−/−^ mice treated with HDM did not show increased Il33 and Il13 protein levels, and only showed a slight increase in CCL11 levels compared to their PBS controls (Figure 3l‐n).

Additionally, to study whether telomerase deficiency also protects from HDM‐induced allergy, a different set of wild‐type and G3 *Tert* ^−/−^ mice, as well as first generation (G1) telomerase‐deficient (G1 *Tert* ^−/−^) mice, were challenged with HDM extract (Figure S1). We found that telomerase deficiency both in G1 and G3 *Tert* ^−/−^ mice attenuates eosinophil and lymphocyte presence in BALF and reduces airway remodeling indicators following HDM exposure (Figure S1a‐j). In accordance with previous results (Figure 3f,o), total lung *Tert* was negligible and *Il13* mRNA expression was highly reduced in G1 and G3 *Tert* ^−/−^ mice compared to *Tert* ^+/+^ mice upon HDM challenge (Figure S1k‐l).

### 2.3 Short telomeres prevent differentiation of Club cells and goblet cell hyperplasia upon HDM‐induced allergy

To further understand the role of telomerase and short telomeres in resistance to HDM‐induced allergy, we analyzed *Tert* mRNA expression levels in total lung extracts (Figure 3o) and measured telomere length in lung sections (Figure 3p,q) in our mouse cohorts. Total lung *Tert* mRNA expression levels were negligible in G3 *Tert* ^−/−^ mice compared to *Tert* ^+/+^ mice both in the PBS‐treated and HDM‐treated lungs. Of interest, *Tert* mRNA was significantly increased in *Tert* ^+/+^ mice upon HDM‐induced allergy compared to PBS‐treated controls (Figure 3o), suggesting that allergic airway inflammation increases *Tert* levels. For telomere length quantification, telomeres were measured in lung sections from *Tert* ^+/+^ and G3 *Tert* ^−/−^ mice by using an immuno‐telomere‐Q‐FISH to evaluate the mean telomere fluorescence (mean telomere spot intensity) and the percentage of short telomeres (corresponding to the 20^th^ percentile of the telomere fluorescence intensity values of controls; PBS‐challenged *Tert* ^+/+^ mice) in bronchial SCGB1A1^+^ Club cells (Figure 3p‐r), which have been previously shown to have a key role in allergic asthma (Sonar et al., 2012). We found that Club cells from G3 *Tert* ^−/−^ lungs showed decreased mean telomere fluorescence and significantly increased percentage of short telomeres compared to wild‐type mice both in the PBS‐treated and HDM‐challenged mice (Figure 3p‐r). In agreement with *Tert* upregulation in wild‐type mice as the consequence of HDM treatment, we also found that telomere fluorescence was significantly increased in wild‐type mice but not in telomerase‐deficient G3 *Tert* ^−/−^ mice after HDM‐induced allergy (Figure 3p,r). Accordingly, we observed a decrease in the percentage of short telomeres in HDM‐challenged *Tert* ^+/+^ mice compared with the PBS controls, which was lower in *Tert* ^−/−^ mice (Figure 3q,r).

Since *Tert* deficiency prevented telomere elongation in Club cells in G3 *Tert* ^−/−^ mice as the consequence of HDM exposure, we next studied the effect of this on the differentiation of Club cells as well as in goblet cell hyperplasia, two phenomena associated with allergic airway inflammation (Figure 3r‐u) (Ren et al., 2013; Tompkins et al., 2009). First, we quantified the number of SCGB1A1^+^ Club cells per epithelium length (mm) as an indication of mucus metaplasia in the bronchi by immunostaining with the Club cell marker SCGB1A1. We found a decreased number of SCGB1A1^+^ Club cells in G3 *Tert* ^−/−^ lungs compared to *Tert* ^+/+^ lungs both in the PBS‐treated and HDM‐challenged lungs (Figure 3r,s). Interestingly, upon HDM exposure, we observed a significant increase in the number of SCGB1A1^+^ Club cells only in *Tert* ^+/+^ mice but not in G3 *Tert* ^−/−^ lungs (Figure 3r,s). Next, we performed double immunostainings with SOX2 (marker of differentiation) and MUC5AC (marker of goblet cells or mucus‐producing cells) in combination with the Club cell marker SCGB1A1 to evaluate the degree of differentiation of Club cells (SCGB1A1^+^‐SOX2^+^ Club cells per epithelium length (mm)) as well as the degree of goblet cell hyperplasia (SCGB1A1^+^‐MUC5AC^+^ cells per epithelium length (mm)) (Figure 3r,t,u). We observed a decrease in the number of SCGB1A1^+^‐SOX2^+^ Club cells in G3 *Tert* ^−/−^ lungs compared to *Tert* ^+/+^ lungs both in the PBS‐treated and HDM‐challenged groups (Figure 3r,t). Again, upon HDM exposure, *Tert* ^+/+^ mice showed a higher increase in the number of SCGB1A1^+^‐SOX2^+^ Club cells compared to similarly treated G3 *Tert* ^−/−^ mice (Figure 3r,t). In addition, HDM‐challenged G3 *Tert* ^−/−^ mice showed less mucus‐producing cells as indicated by a marked reduction in the number of SCGB1A1^+^‐MUC5AC^+^ cells (Figure 3r,u). In agreement with these findings, G3 *Tert* ^−/−^ total lung extracts, showed significantly decreased mRNA expression levels of the goblet cell hyperplasia markers *Foxm1*, *Spdef*, and *Muc5ac* compared with wild‐type lungs, and this difference almost reached statistical significance in the case of *Foxm1* (Figure 3v‐x).

**FIGURE 4:**
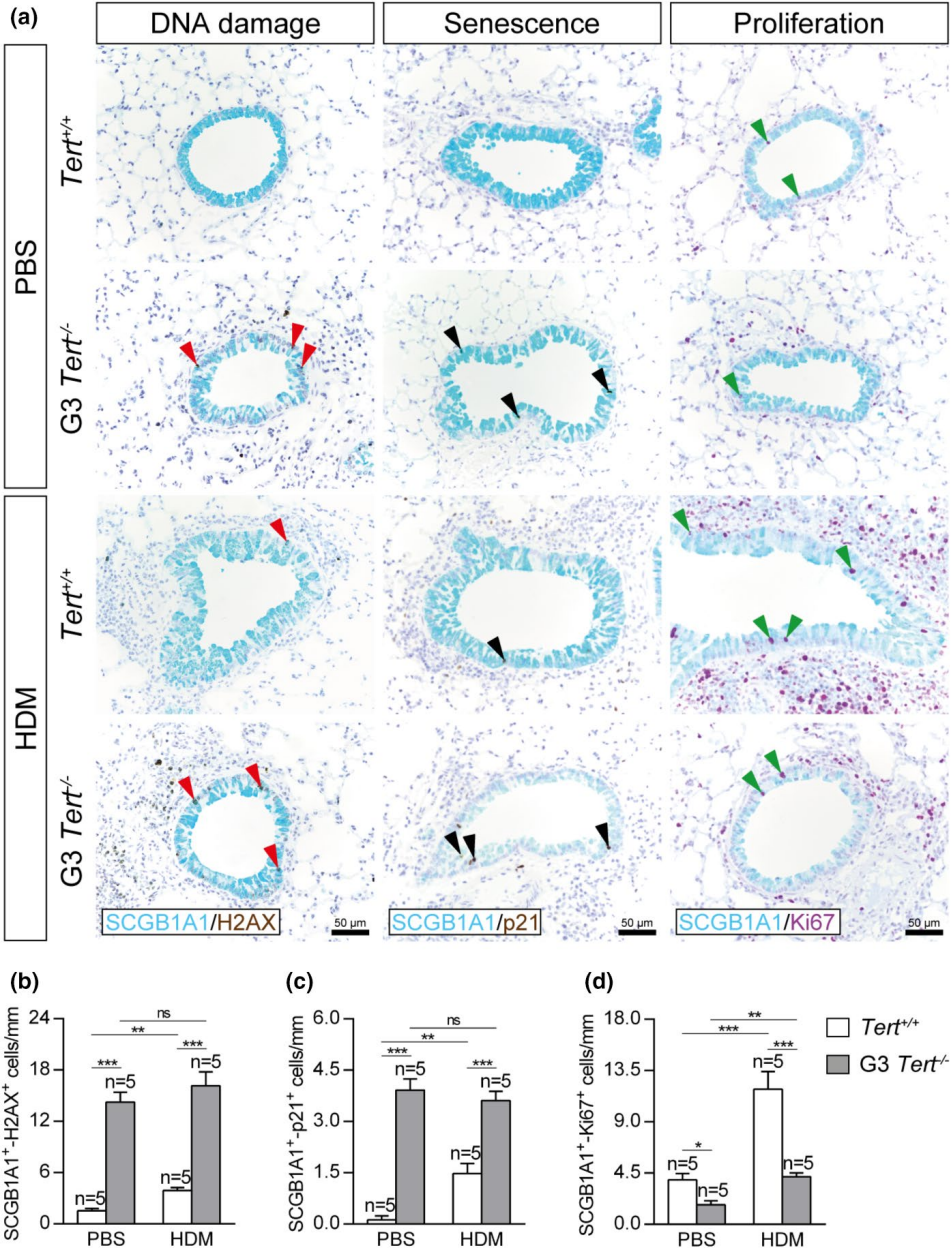
Telomerase deficiency increases DNA damage and senescence and reduces proliferation in Club cells following HDM‐induced allergy. a, Representative images of proximal airways showing immunostainings for SCGB1A1 (blue) and H2AX (brown; red arrowheads indicate double SCGB1A1^+^‐H2AX^+^ Club cells) (left), SCGB1A1 (blue), and p21 (brown; black arrowheads indicate double SCGB1A1^+^‐p21^+^ cells) (center), and SCGB1A1 (blue) and Ki67 (purple; green arrowheads indicate double SCGB1A1^+^‐Ki67^+^ cells) (right) in lung sections from *Tert* ^+/+^ and G3 *Tert* ^−/−^ mice. Quantification of SCGB1A1^+^‐H2AX^+^ (b), SCGB1A1^+^‐p21^+^ (c) and SCGB1A1^+^‐Ki67^+^ (d) Club cells per epithelium length in *Tert* ^+/+^ and G3 *Tert* ^−/−^ mice. Quantifications in lung sections were performed in 4 different bronchi in a random way. Data are expressed as mean ±SEM. **p < 0.01; ***p < 0.001 (Dunn‐Sidak multiple comparison test). The number of mice is indicated in each case

To gain insight into how short telomeres protect from HDM‐induced allergy, we investigated the impact of *Tert* deficiency on DNA damage, senescence, and proliferation of Club cells by performing double immunostainings with H2AX (marker of DNA damage), p21 (marker of senescence) and Ki67 (marker of proliferation) in combination with the Club cell marker SCGB1A1 (Figure 4a‐d). We found an increased number of γH2AX and p21 positive Club cells in both PBS‐ and HDM‐challenged G3 *Tert* ^−/−^ compared to wild‐type mice. Interestingly, no differences in γH2AX and p21 incidence were observed between PBS or HDM‐challenged G3 *Tert* ^−/−^ mice, and we only noticed a moderate increment in these two phenomena in wild‐type mice upon HDM challenge (Figure 4a‐c). Moreover, G3 *Tert* ^−/−^ mice showed a reduced number of SCGB1A1^+^‐Ki67^+^ Club cells as compared to wild‐type PBS‐treated (Figure 4a,d). Notably, upon HDM exposure, an increase in Club cell proliferation was detected in both genotypes that was significantly higher in *Tert* ^+/+^ as compared to G3 *Tert* ^−/−^ mice (Figure 4a,d).

### 2.4 Telomere dysfunction induced by 6‐thio‐dG‐treatment attenuates allergic response upon exposure to HDM

**FIGURE 5:**
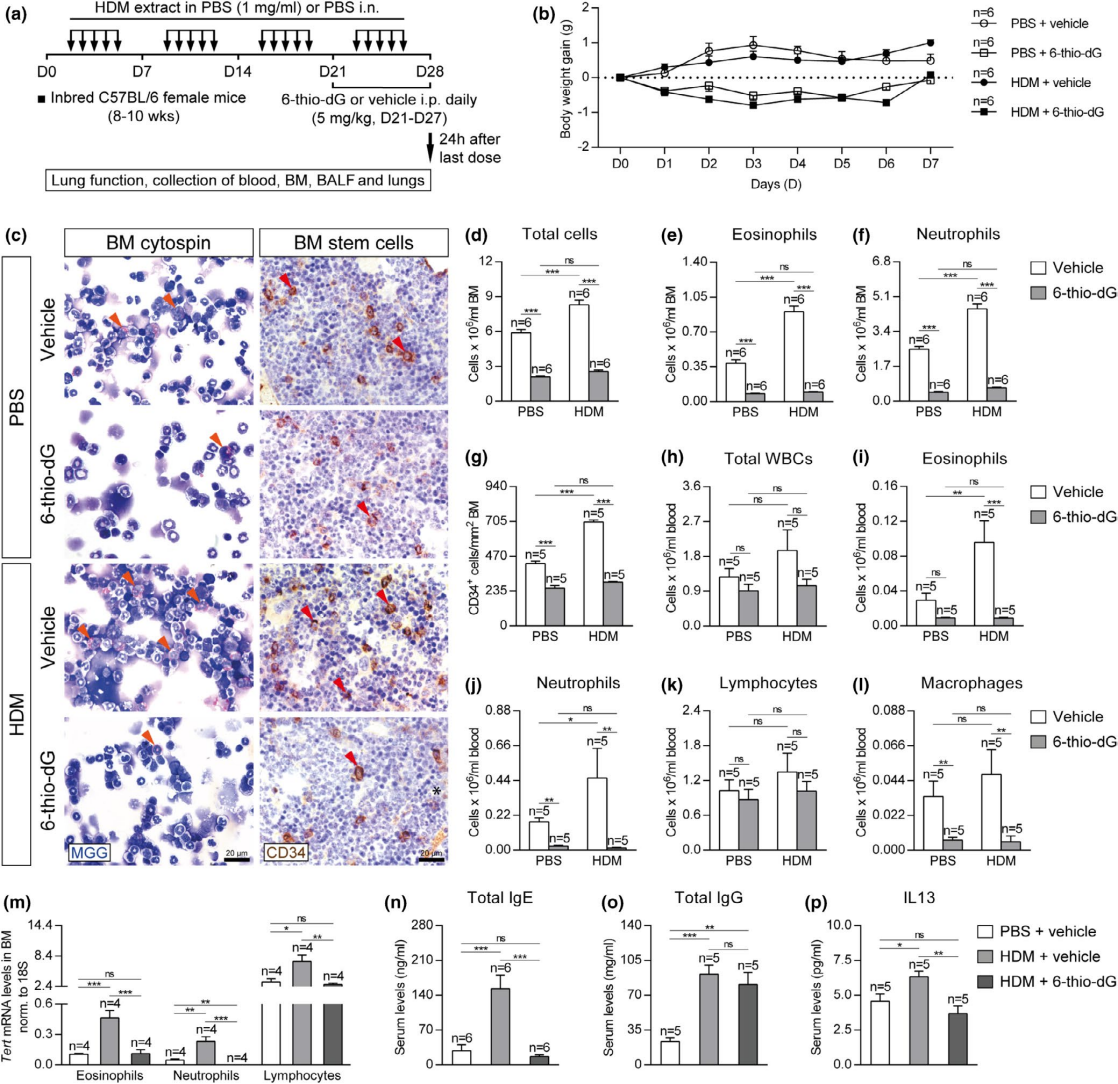
Telomere dysfunction mediated by 6‐thio‐dG attenuates eosinophil and hematopoietic stem cell presence in the bone marrow and depletes serum IgE and IL13 levels and circulating eosinophils after HDM‐induced allergy. a, Eight‐ to 10‐week‐old inbred C57BL/6 female mice were challenged by intranasal administration of 20 μg of house dust mite (HDM) extract in 20 μl of phosphate‐buffered saline (PBS) (1 mg/ml) or equal volume of PBS under inhaled isoflurane anesthesia, five days a week during four weeks. In parallel, the mice were given daily intraperitoneal injections of the telomerase substrate precursor 6‐thio‐dG (5 mg/kg in 5% DMSO) or equal volume of the vehicle during the last week of the HDM protocol (D21‐D27). Lung function assessment and blood, bone marrow (BM), BALF, and lungs were collected 24 h after the last exposure to HDM on day (D) 28. b, Daily follow‐up of the body weight gain in PBS and HDM‐challenged mice treated either with the vehicle or 6‐thio‐dG. c, Representative images showing cytospin preparations (May‐Grünwald Giemsa (MGG) (orange arrowheads indicate eosinophils)) (left) and immunostainings for CD34^+^ hematopoietic stem cells (brown) (red arrowheads) (right) in bone marrow sections from PBS and HDM‐challenged mice treated either with the vehicle or 6‐thio‐dG. Quantification of total cells (d), eosinophils (e), neutrophils (f) and CD34^+^ hematopoietic stem cells (g) in bone marrow sections, and total white blood cells (h), eosinophils (i), neutrophils (j), lymphocytes (k) and macrophages (l) in peripheral blood from PBS and HDM‐challenged mice treated either with the vehicle or 6‐thio‐dG. *Tert* mRNA expression levels in sorted eosinophils, neutrophils, and lymphocytes isolated from the bone marrow of HDM‐challenged mice treated with 6‐thio‐dG vs. controls (m). Quantification of Total IgE (n), Total IgG (o), and IL13 (p) levels in serum from HDM‐challenged mice treated with 6‐thio‐dG vs. controls. Quantifications in bone marrow cytospins and sections were evaluated in 4 different fields in a random way. Data are expressed as mean ±SEM. *p < 0.05; **p < 0.01; ***p < 0.001 (Dunn–Sidak multiple comparison test). The number of mice is indicated in each case

Since short telomeres as the consequence of *Tert* deficiency confer resistance to HDM‐induced allergy, we next set to confirm whether induction of dysfunctional telomeres by using a different approach also leads to allergy resistance. To this end, we induced telomere dysfunction in inbred C57BL/6 mice by administration of the telomerase substrate precursor 6‐thio‐dG, known to induce telomere dysfunction (Mender, Gryaznov, & Shay, 2015), during the last week of the HDM protocol (Figure 5a). We generated four experimental groups: PBS + vehicle (PBS i.n. five days a week for four weeks + 5% DMSO i.p. daily between D21 and D27); PBS + 6‐thio‐dG (PBS i.n. five days a week for four weeks + 5 mg/kg of 6 thio‐dG in 5% DMSO i.p. daily between D21 and D27); HDM + vehicle (HDM i.n five days a week for four weeks + 5% DMSO i.p. daily between D21 and D27); and HDM + 6‐thio‐dG (HDM i.n. five days a week for four weeks + 5 mg/kg of 6 thio‐dG in 5% DMSO i.p. daily between D21 and D27). It should be noted that 6‐thio‐dG‐treated mice showed a slight decrease in the body weight (less than 1 g), which was recovered at the end of the treatment (Figure 5b).

To study the allergic response in these mice, we determined bone marrow cellularity and the number of hematopoietic stem cells in the bone marrows from PBS and HDM‐challenged mice treated either with the vehicle or 6‐thio‐dG (Figure 5c‐g). We found significantly increased total eosinophil and neutrophil counts in HDM‐challenged mice treated with the vehicle compared to their PBS controls, while this increment was not observed in 6‐thio‐dG‐treated mice upon HDM exposure (Figure 5c‐f). Similarly, we observed increased numbers of CD34^+^ hematopoietic stem cells (CD34^+^ cells/mm^2^) upon HDM challenge in the mice treated with the vehicle, while this increment was not found in 6‐thio‐dG‐treated mice (Figure 5c,g). A similar scenario was observed with white blood cell counts. In the case of total white blood cells and lymphocytes, there were no significant changes in total and lymphocyte counts between the treatment groups (Figure 5h,k). However, we observed increased eosinophil and neutrophil counts in the mice treated with the vehicle after HDM‐induced allergy compared to their PBS controls, in contrast to unchanged numbers in 6‐thio‐dG‐treated mice upon HDM exposure (Figure 5i,j). The number of macrophages in blood was found reduced in a similar way in 6‐thio‐dG‐treated mice challenged with both PBS and HDM (Figure 5l). Of note, unchallenged mice treated with 6‐thio‐dG showed a decreased presence of eosinophils and neutrophils in the bone marrow, as well as decreased circulating eosinophil, neutrophil, and macrophage counts (Figure 5c‐l). These observations indicate that 6‐thio‐dG cause a basal deleterious effect on the bone marrow, which would explain the impaired allergic response of 6‐thio‐dG treated mice upon HDM‐induced allergy.

We measured *Tert* mRNA levels in eosinophils, neutrophils, and lymphocytes isolated from the bone marrow of HDM and PBS control mice and in mice treated with 6‐thio‐dG, by fluorescence‐activated cell sorting (FACS) (Figure 5m). We found that *Tert* mRNA expression levels in eosinophils, neutrophils, and lymphocytes were significantly increased in HDM‐treated control mice but not in 6‐thio‐dG‐treated mice compared to PBS‐treated controls. Of note, *Tert* mRNA levels in neutrophils were undetectable in 6‐thio‐dG‐treated mice (Figure 5m).

In particular, serum IgE and IL13 levels were significantly induced in HDM control mice and remained unchanged in 6‐thio‐dG‐treated mice as compared to PBS controls (Figure 5n,p). Serum IgG levels were significantly induced in HDM controls and 6‐thio‐dG‐treated mice in a similar way with respect to PBS controls (Figure 5o).

### 2.5 Telomere dysfunction mediated by 6‐thio‐dG attenuates pulmonary pathology upon HDM‐induced allergy

**FIGURE 6:**
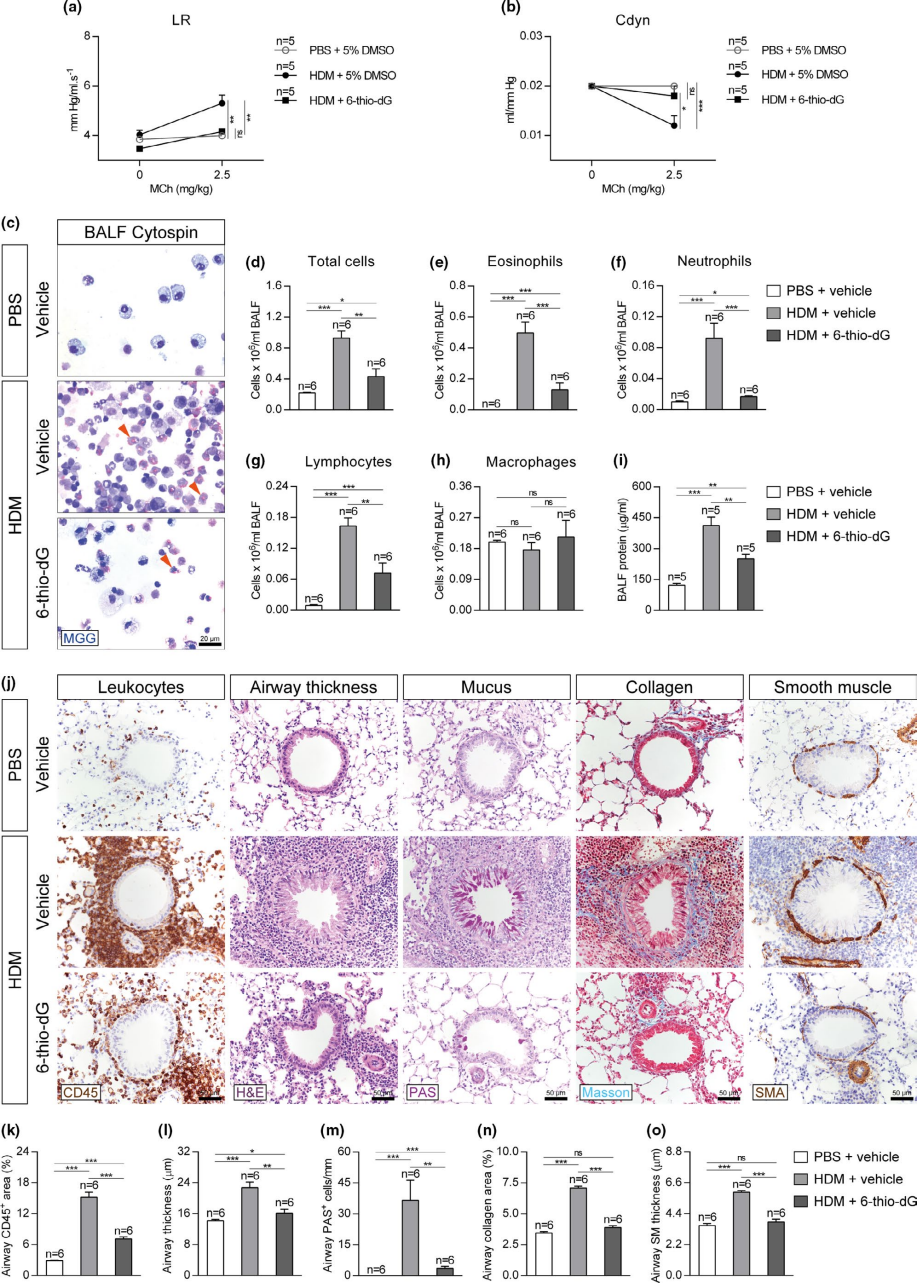
Telomere dysfunction attenuates AHR, eosinophil, and lymphocyte presence in BALF and prevents leukocyte presence and airway remodeling following HDM exposure. Quantification of lung resistance (LR) (a) and dynamic compliance (Cdyn) (b) to methacholine (MCh) evaluated by plethysmography in HDM‐challenged mice treated with 6‐thio‐dG vs. controls. Representative BALF cytospin preparations (May‐Grünwald Giemsa (MGG) (orange arrowheads indicate eosinophils)) (c) and quantification of total (d) and differential BALF cell counts for eosinophils (e), neutrophils (f), lymphocytes (g), and macrophages (h) and total protein concentration in BALF (i) of HDM‐challenged mice treated with 6‐thio‐dG vs. controls. Representative images of proximal airways showing CD45 (brown) and H&E (left), PAS (pink) (center), Masson (blue), and SMA (brown) (right) stainings and immunostainings (j) and quantification of airway CD45^+^ area (leukocytes) (k), airway thickness (l), airway PAS^+^ cells (m), airway collagen area (n) and airway smooth muscle (SM) thickness (o) in lung sections from HDM‐challenged mice treated with 6‐thio‐dG vs. controls. Quantifications in lung sections were performed in 4 different bronchi in a random way. Data are expressed as mean ±SEM. *p < 0.05; **p < 0.01; ***p < 0.001 (Dunn‐Sidak multiple comparison test). The number of mice is indicated in each case

In order to evaluate the effect of telomere dysfunction induced by 6‐thio‐dG on lung function following HDM exposure, airway hyperresponsiveness (AHR) to methacholine was assessed. Of note, HDM‐treated control mice displayed AHR as indicated by increased lung Resistance (LR) and decreased dynamic compliance (Cdyn) with respect to PBS‐treated controls (Figure 6a,b). In contrast, both LR and Cdyn remained unaltered in 6‐thio‐dG‐treated mice (Figure 6a,b).

We further studied the lung phenotypes by evaluating cellularity and total protein levels in bronchoalveolar lavage fluid (BALF). We found increased numbers of total cells and increased BALF cell counts for eosinophils, neutrophils, and lymphocytes in HDM‐treated control mice, while this increment was lower in 6‐thio‐dG‐treated mice with respect to PBS‐treated controls (Figure 6c‐g). In the case of macrophages, there were no significant changes between the treatment groups (Figure 6c,h). Again, total protein levels in BALF were increased in HDM control mice, while this was attenuated in 6‐thio‐dG‐treated mice compared to PBS controls (Figure 6i).

Next, we studied the effect of telomere dysfunction induced by 6‐thio‐dG in the histopathological findings associated with HDM treatment. In particular, we studied several airway remodeling indicators including airway thickness (µm), presence of airway leucocytes (airway CD45^+^ area, (%)), presence of mucus‐producing cells (PAS^+^ cells/mm), airway collagen deposition (airway collagen area (%)), and airway smooth muscle (SM) thickness (µm) (Figure 6j‐o). We found significantly increased CD45^+^ area, airway thickness, and PAS^+^ cells in HDM control mice, while this increment was milder in 6‐thio‐dG‐treated mice compared to PBS‐treated controls (Figure 6j‐m). Finally, airway collagen area and smooth muscle thickness were also incremented in HDM‐treated control mice and remained unaltered in 6‐thio‐dG‐treated mice compared to PBS‐treated controls (Figure 6j,n,o).

**FIGURE 7:**
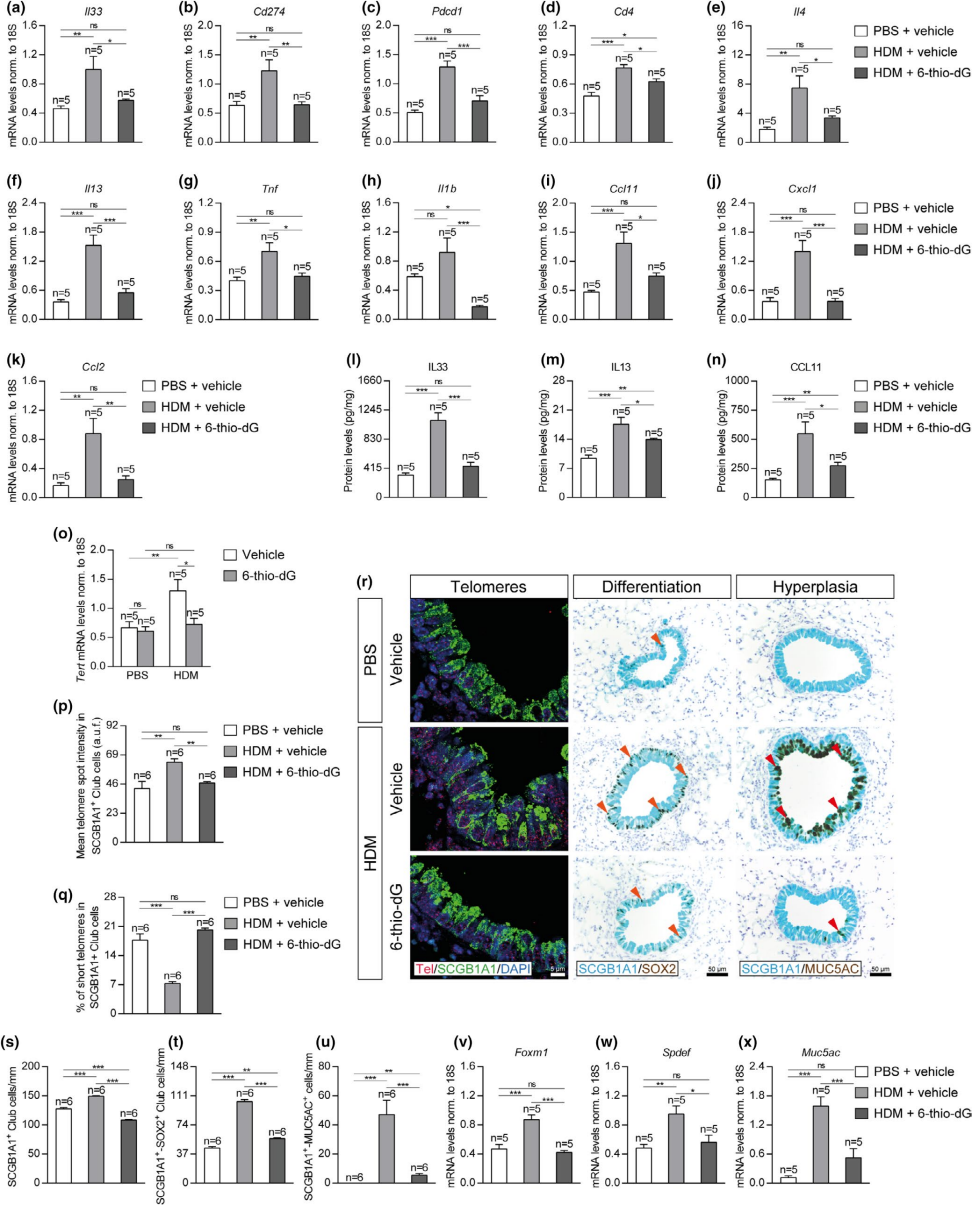
Telomere dysfunction diminishes the expression of allergic markers, prevents telomere elongation and differentiation in Club cells, and attenuates goblet cell hyperplasia after HDM‐induced allergy. (a–k) Lung tissue mRNA expression levels of *Il33* (dendritic cell activation) (a), *Cd274* (PD‐L1) (b) and *Pdcd1* (PD‐1) (c) (Immune checkpoint, T‐cell activation), *Cd4* (T‐cell marker) (d), *Il4* (e) and *Il13* (f) (Th2 cytokines), *Tnf* (g) and *Il1b* (h) (Th1 cytokines), *Ccl11* (eosinophil chemotaxis) (i), *Cxcl1* (neutrophil chemotaxis) (j), and *Ccl2* (macrophage chemotaxis) (k) in HDM‐challenged mice treated with 6‐thio‐dG versus controls. (l‐n) IL33, IL13, and CCL11 protein levels in lung homogenates from HDM‐challenged mice treated with 6‐thio‐dG vs. controls. Lung tissue mRNA expression levels of *Tert* normalized to 18S expression in PBS‐ and HDM‐challenged mice treated with vehicle or 6‐thio‐dG (o). (p‐q) Quantification of mean telomere fluorescence (mean telomere spot intensity) (p) and percentage of short telomeres (q) in Club cells corresponding to the 20^th^ percentil of the fluorescence intensity values of controls (PBS‐challenged mice treated with the vehicle) in HDM‐challenged mice treated with 6‐thio‐dG versus controls. (r) Representative images of proximal airways showing a Q‐FISH for telomere spot fluorescence in Club cells (Cy3Tel probe (red), SCGB1A1^+^ cells (green) and nuclei stained with DAPI (blue)) (left) and representative immunostainings for SCGB1A1 (blue) and SOX2 (brown; orange arrowheads indicate double SCGB1A1^+^‐SOX2^+^ Club cells) (center) and SCGB1A1 (blue) and MUC5AC (brown; red arrowheads indicate double SCGB1A1^+^‐MUC5AC^+^ cells) (right) in lung sections from HDM‐challenged mice treated with 6‐thio‐dG versus controls. (s‐x) Quantification of SCGB1A1^+^ (s) or double SCGB1A1^+^‐SOX2^+^ Club cells (t) and SCGB1A1^+^‐MUC5AC^+^ cells (u) per epithelium length and total lung mRNA expression levels of the goblet cell hyperplasia markers *Foxm1* (v), *Spdef* (w) and *Muc5ac* (x) normalized to 18S expression in HDM‐challenged mice treated with 6‐thio‐dG versus controls. Quantifications in lung sections were performed in 4 different bronchi in a random way. Data are expressed as mean ±SEM. *p < 0.05; **p < 0.01; ***p < 0.001 (Dunn–Sidak multiple comparison test). The number of mice is indicated in each case

Additionally, we measured total lung mRNA expression levels of several allergic airway inflammation markers including *Il33* (dendritic cell activation), *Cd274* (PD‐L1) and *Pdcd1* (PD‐1) (Immune checkpoint, T‐cell activation), *Cd4* (T‐cell marker), *Il4* and *Il13* (Th2 cytokines), *Tnf* and *Il1b* (Th1 cytokines), *Ccl11* (eosinophil chemotaxis), *Cxcl1* (neutrophil chemotaxis), and *Ccl2* (macrophage chemotaxis) (Figure 7a‐k). In general terms, the mRNA expression levels of allergic airway inflammation markers were strongly induced in HDM‐treated control mice and remained unchanged or showed only a mild increase in 6‐thio‐dG‐treated mice compared with PBS‐treated controls, with the exception of *Il1b* which was significantly reduced compared to PBS‐treated control mice (Figure 7a‐k). In agreement with these findings, the protein levels of IL33, Il13, and CCL11 in lung homogenates were significantly increased in HDM‐treated control mice and remained unaltered or only slightly incremented in 6‐thio‐dG‐treated mice compared to PBS‐treated controls (Figure 7l‐n).

### 2.6 Telomere dysfunction induced by 6‐thio‐dG‐treatment prevents Club cell differentiation and Goblet cell hyperplasia upon HDM challenge

Next, we measured total lung *Tert* mRNA levels and telomere length in Club cells in control mice and in mice treated with 6‐thio‐dG (Figure 7o‐r). We found that lung *Tert* mRNA expression levels were significantly increased in HDM‐treated control mice but not in 6‐thio‐dG‐treated mice compared to PBS‐treated controls (Figure 7o). We next measured telomere length in the different mouse cohorts by quantitative telomere FISH and determined both mean telomere fluorescence (mean telomere spot intensity) and the percentage of short telomeres corresponding to the 20^th^ percentile of the telomere fluorescence intensity values of PBS‐treated controls in bronchial SCGB1A1^+^ Club cells (Figure 7p‐r). We found significantly increased mean telomere fluorescence and decreased percentage of short telomeres in HDM‐treated control mice, while these parameters did not vary in 6‐thio‐dG‐treated mice upon HDM challenge compared with PBS‐treated controls (Figure 7p‐r). These findings suggest that telomere dysfunction induced by 6‐thio‐dG, prevents telomere elongation as the consequence of HDM treatment.

Next, we studied the effects of 6‐thio‐dG on allergic airway inflammation, differentiation of Club cells and goblet cell hyperplasia as the consequence of HDM treatment (Figure 7r‐u). First, we quantified the number of Club cells as an indirect measurement of mucus metaplasia in the bronchi by determining the number of SCGB1A1^+^ cells per epithelium length (mm). The number of SCGB1A1^+^ Club cells was significantly increased following HDM exposure in the HDM control mice but reduced in 6‐thio‐dG‐treated mice (Figure 7r‐s). Finally, double immunostainings with the SOX2 differentiation marker and with the MUC5AC marker for goblet cells or mucus‐producing cells were performed in combination with the Club cell SCGB1A1 marker to assess the degree of differentiation of Club cells (SCGB1A1^+^‐SOX2^+^ Club cells per epithelium length (mm)) and goblet cell hyperplasia (SCGB1A1^+^‐MUC5AC^+^ cells per epithelium length (mm)) (Figure 7r,t,u). In particular, the number of SCGB1A1^+^‐SOX2^+^ Club cells and SCGB1A1^+^‐MUC5AC^+^ cells was significantly increased in HDM‐treated control mice but only slightly incremented in 6‐thio‐dG‐treated mice compared to PBS‐treated controls (Figure 7r,t,u). In agreement with these findings, total lung mRNA expression of various goblet cell hyperplasia markers, including *Foxm1*, *Spdef*, and *Muc5ac* were found to be greatly incremented in HDM‐treated control mice but remained largely unaltered in 6‐thio‐dG‐treated mice with respect to PBS‐treated controls (Figure 7v‐x).

**FIGURE 8:**
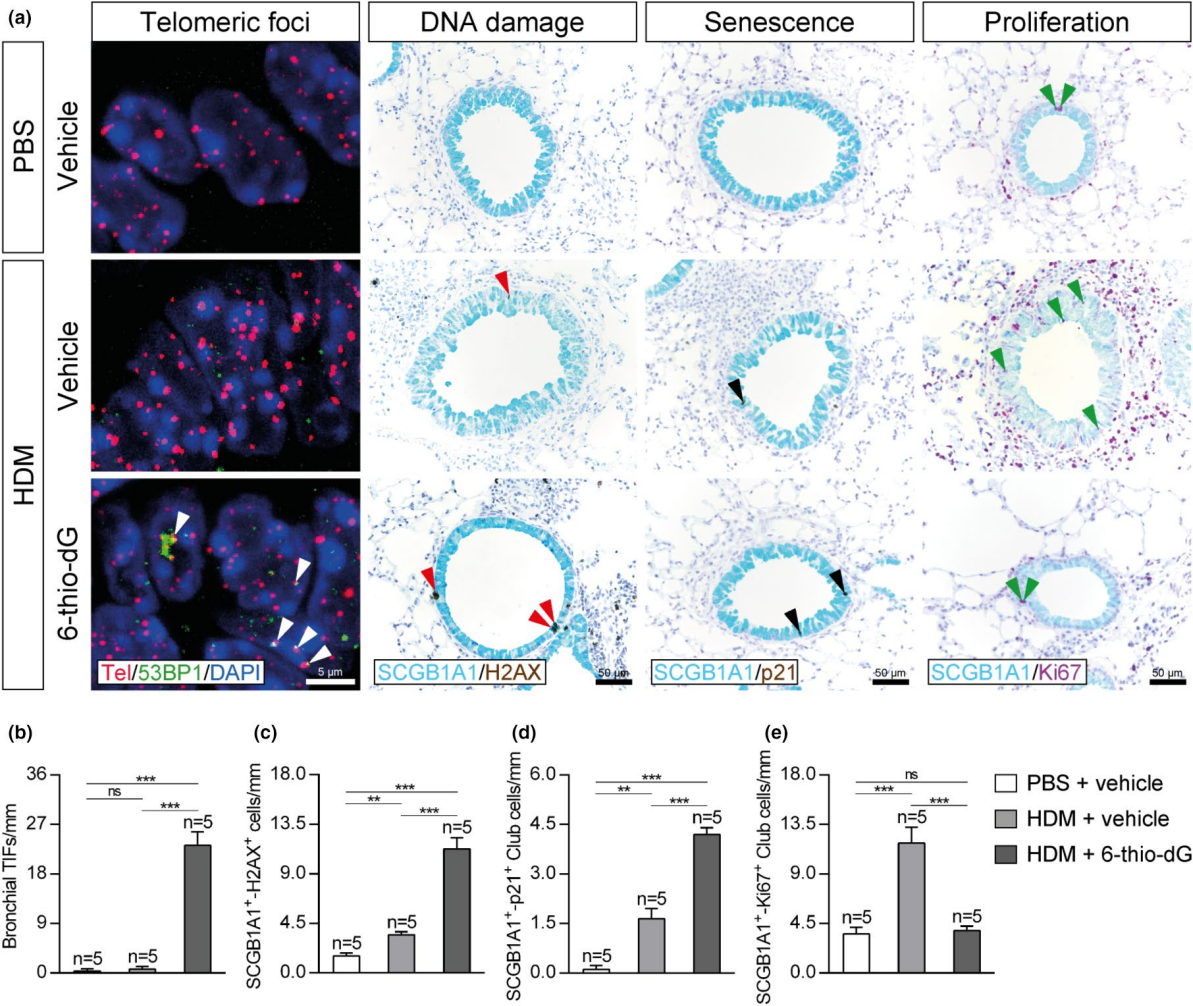
Telomere dysfunction increases bronchial telomeric foci and DNA damage and senescence in Club Cells and prevents increased proliferation in Club cells upon HDM exposure. a, Representative images of proximal airways showing bronchial telomeric induced foci (TIFs) (Cy3Tel probe (red), 53BP1^+^ cells (green; white arrowheads indicate TIFs) and nuclei stained with DAPI (blue)) (left), and immunostainings for SCGB1A1 (blue) and H2AX (brown; red arrowheads indicate double SCGB1A1^+^‐H2AX^+^ Club cells) and SCGB1A1 (blue) and p21 (brown; black arrowheads indicate double SCGB1A1^+^‐p21^+^ cells) (center), and SCGB1A1 (blue) and Ki67 (purple; green arrowheads indicate double SCGB1A1^+^‐Ki67^+^ cells) (right) in lung sections from HDM‐challenged mice treated with 6‐thio‐dG versus controls. Quantification of bronchial TIFs per epithelium length (b), and double SCGB1A1^+^‐H2AX^+^ (c), SCGB1A1^+^‐p21^+^ (d) and SCGB1A1^+^‐Ki67^+^ (e) Club cells per epithelium length in HDM‐challenged mice treated with 6‐thio‐dG versus controls. Quantifications in lung sections were performed in 4 different bronchi in a random way. Data are expressed as mean ±SEM. **p < 0.01; ***p < 0.001 (Dunn‐Sidak multiple comparison test). The number of mice is indicated in each case

To further study, the effects of 6‐thio‐dG on HDM‐induced allergy, we performed an immuno‐telomere‐Q‐FISH with the DNA damage marker 53BP1 in lung tissue sections to evaluate bronchial telomeric induced foci (TIFs) (Figure 8a‐b). We found an increase in bronchial TIFs per epithelium length (mm) in 6‐thio‐dG‐treated mice with respect to HDM and PBS control mice (Figure 8a‐b). Additionally, we investigated the impact of 6‐thio‐dG on DNA damage, senescence, and proliferation of Club cells. We observed an increased number of SCGB1A1^+^‐H2AX^+^ and SCGB1A1^+^‐p21^+^ Club cells per epithelium length (mm) in HDM control mice that was much higher in 6‐thio‐dG‐treated mice with respect to PBS controls (Figure 8a,c,d). Moreover, the increased number of SCGB1A1^+^‐Ki67^+^ Club cells per epithelium length (mm) observed in HDM control mice was not found in 6‐thio‐dG‐treated mice with respect to PBS controls (Figure 8a,e).

## 3 DISCUSSION

Asthma is a chronic inflammatory disease characterized by airway hyperresponsiveness (AHR) and airflow obstruction. As telomere shortening is one of the molecular events associated with organismal aging, we hypothesized here that short telomeres may have an impact on asthma pathobiology. To address this, here we used two independent mouse models with short/dysfunctional telomeres, namely the telomerase‐deficient G1 and G3 *Tert* ^−/−^ mice as well as mice in which we induced a telomere dysfunction by the administration of 6‐thio‐2´‐deoxyguanosine (6‐thio‐dG), and challenged them with HDM extract to induce allergic airway inflammation (Piñeiro‐Hermida, Alfaro‐Arnedo, et al., 2017; Piñeiro‐Hermida, Gregory, et al., 2017). In this regard, it is known that allergen challenges in mice induce responses that are broadly similar to those found in human asthma (Finkelman & Wills‐Karp., 2008; Kumar & Foster., 2012).

6‐thio‐dG is a drug, which is incorporated into telomeric DNA, leading to telomere dysfunction (Mender, Gryaznov, & Shay, 2015). It is worth mentioning that 6‐thio‐dG was reported to cause cell death in telomerase‐expressing cells, but several cell types including human normal fibroblasts, were reported to be unaffected by 6‐thio‐dG (Mender et al., 2015; Sengupta et al., 2018). Specifically, *in vivo* toxicity testing of 6‐thio‐dG at effective doses did not reveal any significant hematologic, renal, or gastrointestinal system rate‐limiting side effects (Mender, Gryaznov, Dikmen, et al., 2015).

In both mouse models, we observed that short/dysfunctional telomeres resulted in a decreased allergic response as indicated by decreased number of CD34^+^ hematopoietic stem cells in the bone marrow. Of note, the decreased allergic response was already observed in G1 *Tert* ^−/−^ mice, suggesting that telomerase deficiency per se may be contributing to hampering the allergic response. In addition, both G3 *Tert* ^−/−^ and 6‐thio‐dG treated mice showed decreased eosinophils in the bone marrow, blood and BALF, being eosinophilia a landmark feature in these cellular compartments upon HDM challenge (Piñeiro‐Hermida, Alfaro‐Arnedo, et al., 2017; Piñeiro‐Hermida, Gregory, et al., 2017).

Lung inflammation in asthma is typically orchestrated by activation of CD4^+^ T lymphocytes which release a wide range of cytokines, to trigger IgE production by B lymphocytes, stimulating the release of inflammatory mediators from immune cells (Busse & Lemansk, 2001; Jacquet, 2011). Indeed, IgE is a well‐known clinical diagnostic biomarker in patients with allergic asthma, previously shown to increase in HDM‐challenged mice (Coverstone et al., 2020; Piñeiro‐Hermida, Alfaro‐Arnedo, et al., 2017; Piñeiro‐Hermida, Gregory, et al., 2017). Interestingly, both G3 *Tert* ^−/−^ and 6‐thio‐dG treated mice showed decreased IgE levels in serum upon HDM challenge, again suggesting an attenuated allergic response as the consequence of telomere dysfunction. Accordingly, *Tert* mRNA levels in bone marrow‐isolated eosinophils, neutrophils, and lymphocytes did not increase upon HDM exposure in 6‐thio‐dG‐treated mice.

In agreement with increased resistance to allergy induced by HDM, both G3 *Tert* ^−/−^ and 6‐thio‐dG treated mice showed improved lung function upon HDM challenge as determined by plethysmography, a well‐known technique for the determination of airway hyperresponsiveness (AHR) in murine models of allergic airway inflammation (Verheijden et al., 2014).

In turn, AHR is dependent on airway remodeling including subepithelial fibrosis, smooth muscle hypertrophy, and goblet cell hyperplasia (Busse, 2010; Evans et al., 2015). In line with this, previous reports have shown increased airway thickness, mucus‐producing cells, airway collagen, and airway smooth muscle thickness in mouse models of HDM‐induced allergy (Piñeiro‐Hermida, Alfaro‐Arnedo, et al., 2017; Piñeiro‐Hermida, Gregory, et al., 2017). Again we show here that all these parameters are significantly decreased in our mouse models of telomere dysfunction, suggesting a hampered allergic response to HDM in the presence of short/dysfunctional telomeres. In particular, HDM‐induced allergy in mice is characterized by Th2 inflammation and epithelial‐to‐mesenchymal transition (EMT) (Heijink et al., 2010). Our results demonstrate that short and dysfunctional telomeres hamper HDM‐induced allergy by reducing the expression of IL33, which is highly produced by bronchial Club cells and reported to regulate EMT and collagen deposition (Sun et al., 2020).

Furthermore, we show here that G3 *Tert* ^−/−^ and 6‐thio‐dG treated mice also showed reduced lung mRNA expression of several allergic airway inflammation markers previously reported to be induced in mice following HDM exposure (Piñeiro‐Hermida, Alfaro‐Arnedo, et al., 2017). Among them, *Cd274* (PD‐L1) and *Pdcd1* (PD‐1) are shown to be important for the activation of T lymphocytes in asthma. Our results showing decreased PD‐L1 and PD‐1 levels in mice with dysfunctional telomeres goes in line with previous findings showing that PD‐L1 and PD1‐deficient mice also show reduced allergic airway inflammation (Akbari et al., 2009; McAlees et al., 2015), as well as with the fact that PD‐1 expression is increased in T‐CD4^+^ lymphocytes of asthmatic patients (Mosayebian et al., 2018). We also find decreased expression of TNF and IL1B that are required for allergen‐specific Th2 cell activation and for the development of AHR in mice (Nakae et al., 2003, 2007). We also show that G3 *Tert* ^−/−^ and 6‐thio‐dG treated mice have reduced expression of the leukocyte marker CD45 in the airways, which is important for the activation of T lymphocytes and eosinophils (Blaylock et al., 2003; Matsuda et al., 1998). Accordingly, telomerase null mice exhibited defective response of the master regulator of inflammation NF‐κB (Ghosh et al., 2012), which is consistent with our results.

In line with a decreased inflammatory response as the consequence of short telomeres, we also observed decreased protein levels of IL33, IL13, and CCL11 in the lungs of G3 *Tert* ^−/−^ and 6‐thio‐dG treated mice. IL33 is a central activator of dendritic cells to induce Th2 immunity during HDM allergic sensitization (Chu et al., 2013; Makrinioti et al., 2014) and was previously shown to exacerbate murine allergic bronchoconstriction (Sjöberg et al., 2015). IL13 is a central mediator of allergic asthma and its blockade in mice upon HDM exposure reduces eosinophilia in BALF, peribronchial collagen, and goblet cell hyperplasia (Tomlinson et al., 2010). Finally increased expression of CCL11 in the bronchial epithelium of asthmatic patients is associated with AHR (Ying et al., 1997). CCL11 is released by bronchial Club cells in response to cytokines such as IL4, IL13, and TNF and it is important for the accumulation of eosinophils during allergic lung inflammation (Conroy & Williams, 2001; Sonar et al., 2012).

We demonstrate that Club cells of G3 *Tert* ^−/−^ mice present shorter telomeres. Accordingly, human bronchial epithelial cells (HBEC) divided for over 200 population doublings exhibited shorter telomeres and senescence (Peters‐Hall et al., 2020). In addition, we recently reported that telomerase deficiency in mice leads to telomere shortening in Club cells (Piñeiro‐Hermida et al., 2020). Remarkably, short and dysfunctional telomeres prevented differentiation in Club cells and goblet cell hyperplasia upon HDM‐induced allergy. In this respect, short telomeres have been previously shown to impair stem cell function (Flores et al., 2005; Martínez & Blasco, 2011). Specifically, Club cells were reported to play a major role in allergic asthma (Sonar et al., 2012) and SOX2 was reported to be required for goblet cell differentiation after allergen sensitization (Tompkins et al., 2009). On the other hand, upon allergen stimulation, FOXM1 induces differentiation of Club cells into goblet cells through transcriptional activation of SPDEF. Then, increased MUC5AC expression by SPDEF in goblet cells contributes to mucus hyperproduction and AHR (Chen et al., 2009; Ren et al., 2013). In this sense, blockade of FOXM1 activity in mice after HDM exposure led to reduced goblet cell hyperplasia (Sun et al., 2017). Moreover, SPDEF and MUC5AC‐deficient mice were reported to show improved lung function upon allergic airway inflammation (Evans et al., 2015; Rajavelu et al., 2015).

Telomeric dysfunction mediated by 6‐thio‐dG caused an increase in bronchial TIFs, DNA damage, and senescence in Club cells, phenomena already reported in tumor cells treated with 6‐thio‐dG (Mender, Gryaznov, Dikmen, et al., 2015; Sengupta et al., 2018). In agreement with previous results, we show that G3 *Tert* ^−/−^ mice presented increased DNA damage and senescence, and decreased proliferation in Club cells (Piñeiro‐Hermida et al., 2020). The increment in senescence, DNA damage, and proliferation in Club Cells by HDM challenge has also been observed in the bronchi of several asthma mouse models (Wu et al., 2013; Chan et al., 2016; Tam et al., 2019).

We show that short telomeres hamper asthma development and this might explain at least in part the previously reported age‐related asthma incidence decrease (Dharmage et al., 2019; Pakkasela et al., 2020). In summary, our findings imply that short/dysfunctional telomeres play a relevant role in allergen‐induced airway inflammation, mediating both AHR and mucus secretion after HDM‐induced allergy.

## 4 MATERIALS AND METHODS

### 4.1 Ethical statement

All experiments and animal procedures were approved by our Institutional Animal Care and Use Committee (IACUC) and by the Ethics Committee for Research and Animal Welfare (CEIyBA).

### 4.2 Mice and HDM‐induced allergic inflammation

*Tert* heterozygous mice were generated as previously described (Liu et al., 2000) and backcrossed to >98% C57BL/6 background. *Tert* ^+/+^ and first (G1) and third (G3) generation *Tert* ^−/−^ female mice were generated as illustrated in Figure 1a and intranasally challenged with 20 μg of HDM extract (Greer Laboratories Inc, Lenoir, NC) or PBS for four weeks (Piñeiro‐Hermida, Alfaro‐Arnedo, et al., 2017; Piñeiro‐Hermida, Gregory, et al., 2017). Lung function assessment and collection of samples were performed on day 28 (Figure 1b). Additionally, inbred C57BL/6 female mice were challenged with HDM extract or PBS along with daily intraperitoneal injections of 6‐thio‐dG (5 mg/kg) or vehicle during the last week of the HDM protocol (D21‐D27) (Figure 5a,b).

### 4.3 In vivo measurement of lung function

The mice were anesthetized using 10 μl/g of ketamine‐medetomidine, intubated with a 24‐gauge catheter (BD, Franklin lakes, NJ, USA) and intravenously injected with 2.5 mg/kg of methacholine (MCh) (Sigma‐Aldrich, St. Louis, MO, USA) (Zoltowska Nilsson et al., 2018). Lung function was assessed in a plethysmograph (SCIREQ, Montreal, Canada) for the determination of LR (lung resistance) and Cdyn (dynamic compliance).

### 4.4 Sample collection and processing

Animals were euthanized using 10 μl/g of ketamine‐xylazine. Blood was collected by cardiac puncture and lungs were lavaged with cold PBS 1X. Right lung lobes were dissected and snap‐frozen in liquid nitrogen for quantitative PCR (qPCR) and ELISA analyses, and the left lung lobe was harvested for histopathological evaluation or immunohistochemistry. Femurs were dissected for histopathology and immunohistochemistry and to perform bone marrow cytospin preparations (Piñeiro‐Hermida, López, et al., 2017).

### 4.5 Histopathological analyses and immunostaining

Hematoxylin and eosin (H&E) staining was performed for the quantification of airway thickness, and Periodic acid‐Schiff (PAS) and Masson´s trichrome (DAKO, Agilent technologies, Santa Clara, CA) stainings to evaluate the number of mucus‐producing cells and the degree of collagen deposition. Immunostainings were performed using the following antibodies: CD34 (Clone RAM34 1:100, Invitrogen, Carlsbad, CA), Ki‐67 (Clone D3B5 1:50, Cell Signaling Technology, Danvers, MA), CD45 (Clone D3F8Q 1:500, Cell signaling technology), SMA (Clone 1A4 1:4, DAKO, Agilent technologies, Santa Clara, CA), CC10 (Clone T‐18 1:1000, Santa Cruz Biotechnology), SOX2 (Clone C70B1 1:75, Cell Signaling Technology), MUC5AC (Clone 45 M1 1:50, Thermo Fisher Scientific, Waltham, MA), H2AX (Ser139, Clone JBW301 1:200, EMD Millipore, Burlington, MA), p21 (Clone 291H/B5 1:10, CNIO Monoclonal Antibodies Core Unit, Madrid, Spain), and Ki67 (Clone D3B5 1:50, Cell Signaling Technology). CD34 and Ki67 antibodies were used for the quantification of hematopoietic stem cells and proliferation in the BM. CD45 and SMA antibodies served to quantify airway leukocytes and smooth muscle thickness. CC10, SOX2, and MUC5AC antibodies were used to evaluate the number and differentiation of Club cells, and for the assessment of goblet cell hyperplasia.

### 4.6 Telomere Q‐FISH analyses

After deparaffinization and rehydration, tissues underwent antigen retrieval in 10 mM sodium citrate buffer, and permeabilization was performed in PBS 0.5% Triton X‐100 for 3 hours. Next, tissues were washed 3x5 min in PBS 1X, fixed in 4% formaldehyde for 5 min, washed 3 × 5 min in PBS and dehydrated in a 70%–90%–100% ethanol series (5 min each). The immuno‐telomere quantitative FISH (Q‐FISH) in SCGB1A1‐positive Club cells was performed and analyzed as previously described (Piñeiro‐Hermida et al., 2020). Following the same protocol, an immuno‐telomere‐Q‐FISH with the DNA damage marker 53BP1 (1:500, Novus Biologicals, Centennial, CO) was also performed.

### 4.7 RNA isolation, reverse transcription and qPCR

Inferior right lung lobes were homogenized in TRIzol reagent (Invitrogen, Carlsbad, CA), and RNA was isolated using an RNeasy Mini Kit (Qiagen, Hilden, Germany) and reverse‐transcribed to cDNA using SuperScript II First‐Strand Synthesis System (Invitrogen). qPCR was performed as previously described (Piñeiro‐Hermida et al., 2020). Primer sets used for qPCR are included within the Table S1.

### 4.8 Fluorescence‐activated cell sorting (FACS)

Following preincubation with anti‐CD16/CD32 (clone 2.4G2 1:200, BD), BM cells were incubated with the following antibodies for 30 min at 4°C: CD11b (Clone M1/70 1:200, FITC, eBioscience, Sand Diego, CA); Ly6G (Clone 1A8 1:200, PerCP‐Cy5.5, BioLegend, San Diego, CA); Siglec‐F (Clone E50‐2440 1:200, APC‐Cy7, BD); CD3 (Clone 17A2 1:200, PE, BioLegend, San Diego, CA) and CD4 (Clone RM4.5 1:400, PE‐Cy7, BD). Then, cells were washed, resuspended in FACS buffer and stained with DAPI. Eosinophils, neutrophils, and CD4 T lymphocytes were sorted using a FACSAria III sorter (BD) in TRIzol LS reagent (Invitrogen), and RNA was isolated using the 5PRIME Phase Lock gel columns (Quantabio, Beverly, MA) and the PicoPure RNA Isolation Kit (Thermo Fisher Scientific). Reverse transcription to cDNA and qPCR were performed as described above.

### 4.9 ELISAS

Serum total IgE and IgG levels were assessed with IgE and IgG mouse ELISA kits (Abcam, Cambridge, UK). Cytokine levels were assessed in homogenized lung tissue lysates using mouse IL13 Duoset and IL33 and CCL11 Quantikine ELISA Kits (R&D systems, Minneapolis, MN) and normalized to total lung protein levels.

### 4.10 Statistics

Following a Shapiro–Wilk normality test, either a One‐way ANOVA test or a Kruskal–Wallis test were used, and then, the post hoc Dunn‐Sidak test was carried out for multiple comparisons. Results are shown as mean values ±standard error of the mean (SEM).

## CONFLICT OF INTEREST

There is no conflict of interest to declare.

## AUTHOR CONTRIBUTIONS

M.A. Blasco had the original idea and secured funding. M.A. Blasco and P. Martínez, supervised research. M.A. Blasco, P. Martínez and S. Piñeiro‐Hermida wrote the paper. S. Piñeiro‐Hermida analyzed the data and performed experiments.

## Supporting information

Supplementary Material

Figure S1

## Data Availability

The data that support the findings of this study are available from the corresponding author upon reasonable request.
