# Supplementary material for: Short and dysfunctional telomeres protect from allergen‐induced airway inflammation
Source: Aging Cell. 2021 May 4;20(5):e13352. doi: 10.1111/acel.13352 (PMC8135011; doi:10.1111/acel.13352)
Supplement: Supplementary file 1 — Supplementary Material [file ACEL-20-e13352-s001.docx]

***Supporting information for***

**Short and dysfunctional telomeres protect from allergen-induced airway inflammation**

**Sergio Piñeiro-Hermida^1^, Paula Martínez^1^ and Maria A. Blasco^1,#^**

^1^Telomeres and Telomerase Group, Molecular Oncology Program, Spanish National Cancer Centre (CNIO), Melchor Fernández Almagro 3, Madrid, E-28029, Spain.

**Files included:**

**Supplementary methods**

**Table S1**. Primer sets used for qPCR

Figure S1. Telomerase deficiency in G1 and G3 *Tert^-/-^* mice attenuates eosinophil and lymphocyte presence in BALF and reduces airway remodeling following HDM exposure

**Supplementary methods**

**Ethical statement**

The animals were bred and maintained under specific pathogen-free (SPF) conditions in laminar flow caging at the CNIO animal facility in accordance with the recommendations of the Federation of European Laboratory Animal Science Associations (FELASA).

**Mice and HDM-induced allergic inflammation**

*Tert^+/-^* mice were intercrossed to generate *Tert^+/+^* and first generation (G1) homozygous *Tert^-/-^* mice. Second generation (G2) *Tert^-/-^* mice were generated by successive breeding of G1 *Tert^-/-^* and then third generation (G3) *Tert^-/-^* mice by crosses between G2 *Tert^-/-^* mice (Figure 1A). Eight- to 10-week-old female *Tert^+/+^*, and G1 and G3 *Tert^-/-^* mice were intranasally (i.n.) challenged with 20 μg of HDM extract in 20 μl of PBS (1 mg/ml) or equal volume of PBS under light isoflurane anesthesia, five days a week for four weeks. Lung function assessment and blood, bone marrow (BM), BALF and lungs were collected 24h after the last exposure to HDM on day 28 (Figure 1B). Additionally, the same protocol for the induction of allergic airway inflammation was performed using inbred C57BL/6 female mice and in parallel, the mice were given daily intraperitoneal injections of the telomerase substrate precursor 6-thio-dG (5 mg/kg in 5% DMSO) or equal volume of the vehicle during the last week of the HDM protocol (D21-D27) (Figure 4A).

**In vivo measurement of lung function**

In vivo measurement of lung function was performed 24h after the last HDM exposure. The mice were anesthetized by intraperitoneal injection of 10 μl/g of a ketamine-medetomidine anesthetic combination in saline (75:1 mg/kg respectively). The mice received a single intravenous tail injection of 2.5 mg/kg of metacholine and lung function was assessed within 5 minutes in a plethysmograph having previously measured the basal lung function. A MiniVent (Harvard Apparatus, Holliston, MA, USA) was connected to the plethysmograph and the tracheal cannula for animal ventilation at 10 ml/kg of tidal volume and 150 breaths per minute. Data were measured by 2 pressure transducers that detect pressure variations in the chamber (flow) and in the tracheal cannula (pressure).

**Sample collection and processing**

Animals were euthanized by intraperitoneal injection of 10 μl/g of a ketamine-xylazine anesthetic combination in saline (100:10 mg/kg respectively) after lung function assessment.

Serum was obtained by centrifugation at 3000 xg for 10 min at 4 °C and stored at -80°C until further usage and lungs and decalcified femurs were formalin-fixed and embedded in paraffin. On the other hand, bronchoalveolar lavage fluid (BALF) was centrifuged at 10000 rpm for 5 min at 4 °C and the supernatants were stored at -80 °C to subsequently assess total protein concentration in BALF using the Pierce BCA Protein Assay Kit (Thermo Fisher Scientific, Waltham, MA, USA). Hereafter, the BALF pellets were resuspended in 500 μl of ACK Lysing Buffer (Thermo Fisher Scientific) and centrifuged at 10000 rpm for 5 min at 4 °C after 10 min of incubation. The supernatants were discarded and 500 µl of PBS 1X were added to the pellet to prepare the cytospin preparations by centrifugation of the slides at 1500 rpm for 5 min.

The femoral bone marrow (BM) isolation was performed by dissection of the femoral heads. After centrifugation at 10000 xg for 15 seconds, BM was suspended in 500 μl PBS 1X and centrifuged at 300 xg for 5 min at 4 °C. Following aspiration of the supernatants, BM pellets were resuspended in 500 μl of ACK Lysing Buffer (Thermo Fisher Scientific) and centrifuged at 300 xg for 5 min at 4 °C after 10 min of incubation. The supernatants were discarded and 1 ml of PBS 1X was added to the pellet to prepare the cytospin preparations by centrifugation of the slides at 1500 rpm for 5 min.

**Quantification of BALF and bone marrow**

Total cell number was counted and expressed as cells/ml of BALF or bone marrow, and differential cell counts were performed on May-Grünwald Giemsa (Sigma-Aldrich)-stained cytospins, counting a minimum of 300 cells per slide or 4 different fields per slide in BALF and bone marrow cytospins, respectively. Determination of differential cell counts was performed using standard morphology criteria.

**Histopathological analyses and immunostaining**

Paraffin-embedded lungs were cut into 3 μm sections for histopathological evaluation or immunohistochemistry. Fiji open source image processing software package v1.48r (http://fiji.sc) was used for the quantification of Ki67^+^ and CD45^+^ areas (percentage of DAB), airway collagen area, airway and smooth muscle thickness and epithelium length measurements. Quantifications in bone marrow and lung sections were performed in 4 different fields or bronchi, respectively in a random way.

**Fluorescence-activated cell sorting (FACS)**

BM pellets obtained as described above, were resuspended in 1 ml of FACS buffer (3 mM EDTA, 0.1% BSA in PBS 1X) and centrifuged at 300 xg for 5 min at 4 °C. The supernatants were discarded and cell suspensions were preincubated for 10 min with anti-CD16/CD32 to reduce non-specific binding of fluorescent antibodies. Eosinophils (CD11b^+^, Ly6G^-^, Siglec-F^+^), neutrophils (CD11b^+^, Ly6G^+^, Siglec-F^-^) and CD4 T lymphocytes (CD3^+^, CD4^+^) where sorted using a FACSAria III sorter (BD).

**ELISAS**

Superior right lung lobes were homogenized in RIPA Buffer (Thermo Fisher Scientific) containing a protease-phosphatase inhibitor mixture (Roche, Basel, Switzerland), and total protein concentration was determined with the Pierce BCA Protein Assay Kit (Thermo Fisher Scientific).

**Statistics**

Statistical analyses were accomplished using SPSS Statistics Software v21 for Windows (IBM, Armonk, NY, USA). For all analyses, a p value<0.05 was considered statistically significant.

**Table S1.** Primer sets used for qRT-PCR.

| **Gene** | **Accession No.** | **Forward primer (5´-3´)** | **Reverse primer (5´-3´)** |
| --- | --- | --- | --- |
| *Ccl2* | NM_011333.3 | CACCAGCCAACTCTCACTGA | CGTTAACTGCATCTGGCTGA |
| *Ccl11* | NM_011330.3 | GAGAGCCTACAGAGCCCAGA | ACCGTGAGCAGCAGGAATAG |
| *Cd4* | NM_013488.2 | ATGTGGAAGGCAGAGAAGGA | TGGGGTATCTTGAGGGTGAG |
| *Cd274* | NM_021893.3 | CATACCGCAAAATCAACCAG | CACTTCTCTTCCCACTCACG |
| *Cxcl1* | NM_008176.3 | ATCCAGAGCTTGAAGGTGTTG | GTCTGTCTTCTTTCTCCGTTACTT |
| *Foxm1* | NM_008021.4 | CCTGCTTACTGCCCTTTCCT | CACACCCATCTCCCTACACC |
| *Il1b* | NM_008361.3 | GCAACTGTTCCTGAACTCAACT | ATCTTTTGGGGTCCGTCAACT |
| *Il4* | NM_021283.2 | CCTCACAGCAACGAAGAACA | CGAAAAGCCCGAAAGAGTC |
| *Il13* | NM_008355.3 | GCCTCCCCGATACCAAAAT | CTTCCTCCTCAACCCTCCTC |
| *Il33* | NM_133775.2 | GCCTTGCTCTTTCCTTTTCTC | TCGGTTGTTTTCTTGTTTTGC |
| *Muc5ac* | NM_010844.1 | CACACACAACCACTCAACCA | TCTCTCTCCGCTCCTCTCAA |
| *Pdcd1* | NM_008798.2 | TCAAGGCATGGTCATTGGTA | GCTCCTCCTTCAGAGTGTCG |
| *Rn18s* | NR_003278.3 | ATGCTCTTAGCTGAGTGTCCCG | ATTCCTAGCTGCGGTATCCAGG |
| *Spdef* | NM_013891.4 | GGCCAGCCATGAACTATGAT | GGTAGACAAGGCGCTGAGAG |
| *Tert* | NM_009354.2 | TGACCAGCGTGTTAGGAAGA | CAGGAGGAAAGGAGCCAGAG |
| *Tnf* | NM_013693.3 | GCCTCTTCTCATTCCTGCTTG | CTGATGAGAGGGAGGCCATT |

| **Figure S1.** Telomerase deficiency in G1 and G3 *Tert^-/-^* mice attenuates eosinophil and lymphocyte presence in BALF and reduces airway remodeling following HDM exposure.  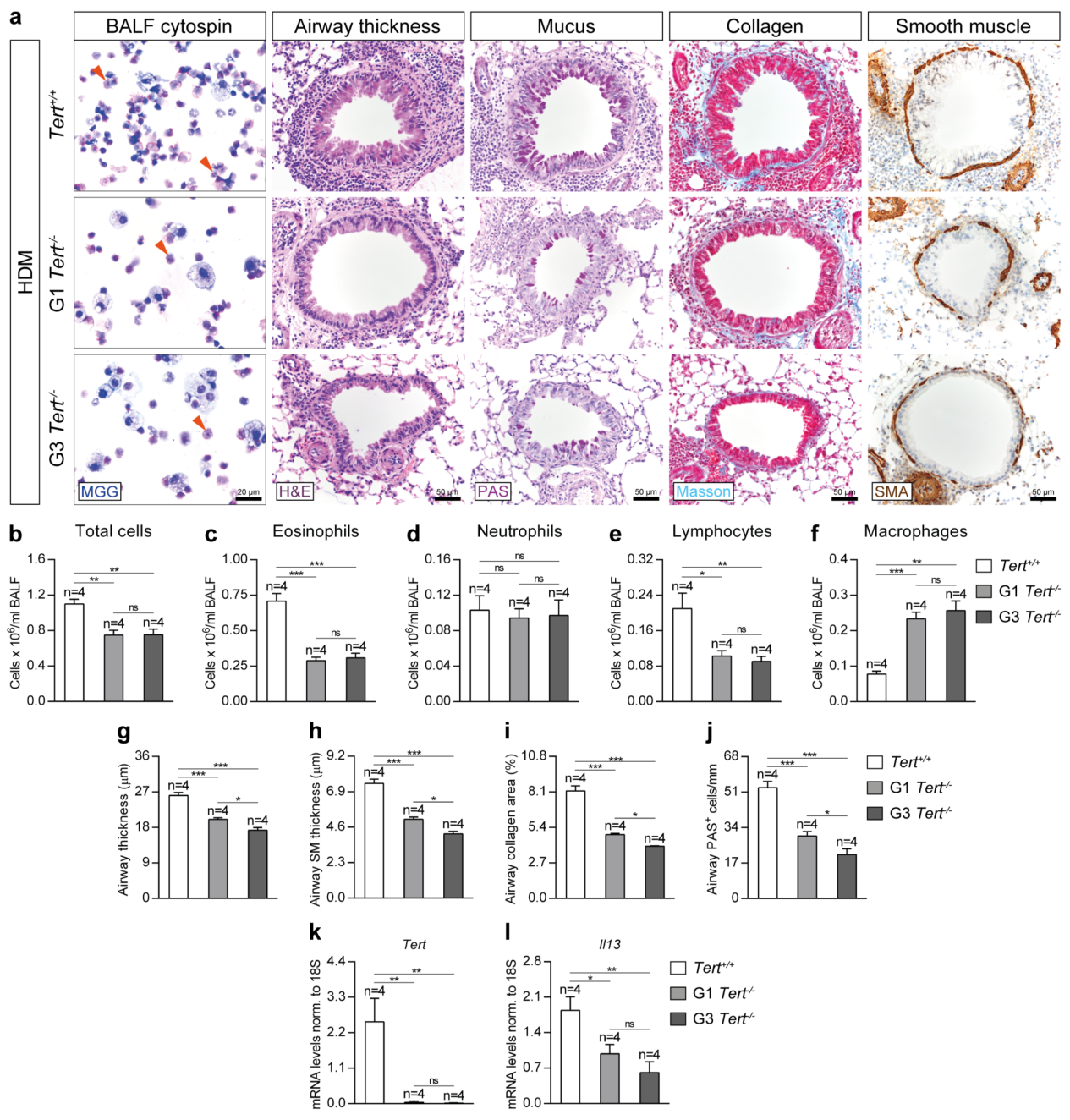 |
| --- |
| **Figure legend.** Telomerase deficiency in G1 and G3 *Tert^-/-^* mice attenuates eosinophil and lymphocyte presence in BALF and reduces airway remodeling following HDM exposure. Representative BALF cytospin preparations (May-Grünwald Giemsa (MGG) (orange arrowheads indicate eosinophils)), and images of proximal airways showing H&E, PAS (purple), Masson (blue) and SMA (brown) stainings and immunostainings (a), and quantification of total (b) and differential BALF cell counts for eosinophils (c), neutrophils (d), lymphocytes (e) and macrophages (f) in *Tert^+/+^* and G1 and G3 *Tert^-/-^* mice. Quantification of airway thickness (g), airway PAS^+^ cells (h), airway collagen area (i) and airway smooth muscle (SM) thickness (j) in lung sections from *Tert^+/+^* and G1 and G3 *Tert^-/-^* mice. Total lung mRNA expression levels of *Tert* (k) and *Il13* (l) normalized to 18S expression in *Tert^+/+^* and G1 and G3 *Tert^-/-^* mice. Quantifications in lung sections were performed in 3 different bronchi in a random way. Data are expressed as mean ± SEM. *p<0.05; **p<0.01; ***p<0.001 (Dunn-Sidak multiple comparison test). The number of mice is indicated in each case. |
