# Supplementary figures and images for: Short and dysfunctional telomeres protect from allergen‐induced airway inflammation
Source: Aging Cell. 2021 May 4;20(5):e13352. doi: 10.1111/acel.13352 (PMC8135011; doi:10.1111/acel.13352)

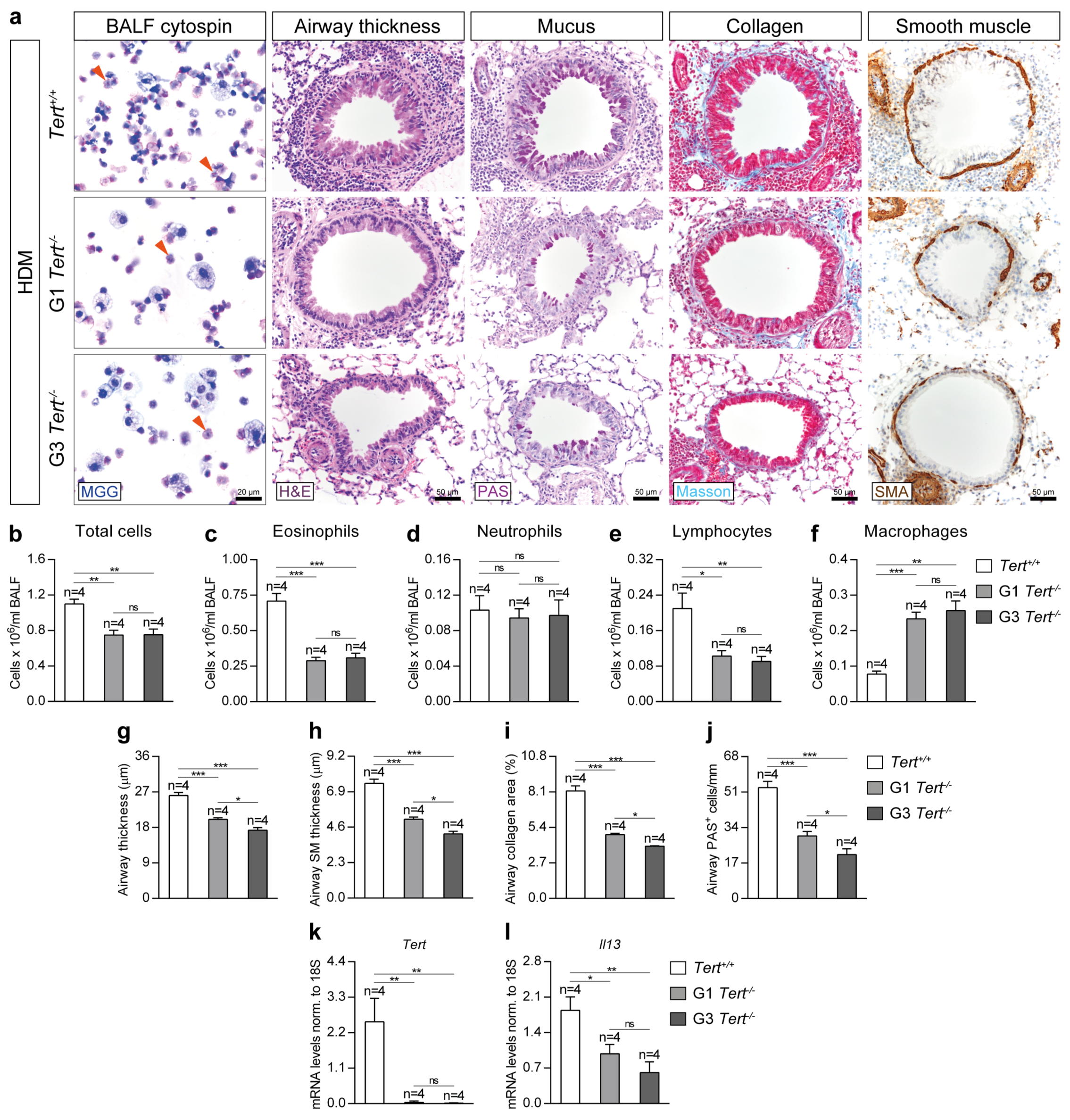

Supplement: Supplementary file 2 — Figure S1 [file ACEL-20-e13352-s002.tif]
